# Machine learning-based prediction of antimicrobial resistance and identification of AMR-related SNPs in *Mycobacterium tuberculosis*

**DOI:** 10.1186/s12863-025-01338-x

**Published:** 2025-07-12

**Authors:** Yi Xu, Ying Mao, Xiaoting Hua, Yan Jiang, Yi Zou, Zhichao Wang, Zubi Liu, Hongrui Zhang, Lingling Lu, Yunsong Yu

**Affiliations:** 1 Department of Infectious Diseases, Sir Run Run Shaw Hospital, Zhejiang University School of Medicine, Hangzhou, Zhejiang 310016 China; 2 Institute of Translational Medicine, Zhejiang University School of Medicine, Hangzhou, Zhejiang 310029 China; 3 Key Laboratory of Digital Technology in Medical Diagnostics of Zhejiang Province, Dian Diagnostics Group Co, Ltd, Hangzhou, 310030 China; 4 Key Laboratory of Microbial Technology and Bioinformatics of Zhejiang Province, Hangzhou, 310016 China; 5 Department of Clinical Laboratory, Zhejiang Hospital, Hangzhou, Zhejiang 310013 China

**Keywords:** Antimicrobial resistance (AMR), *M. tuberculosis* (MTB), Machine learning (ML), Whole genome sequencing (WGS), Single nucleotide polymorphisms (SNPs)

## Abstract

**Background:**

*Mycobacterium tuberculosis* (MTB) is a human-specific pathogen that primarily infects humans, causing tuberculosis (TB). Antimicrobial resistance (AMR) in MTB presents a formidable challenge to global health. The employment of machine learning on whole-genome sequencing data (WGS) presents significant potential for uncovering the genomic mechanisms underlying drug resistance in MTB.

**Methods:**

We used 18 binary matrices, each consisting of genotypes and antimicrobial susceptibility testing phenotypes from a specific MTB-antimicrobial dataset. By constructing training and test datasets on all SNPs, intersected SNPs, and randomly generated SNPs, we developed a Machine learning (ML) framework using twelve different algorithms. Then, we compared the performances of the various ML models and used the SHapley Additive exPlanations (SHAP) framework to decipher why and how decisions are made within the optimal algorithm. Lastly, we applied the models to predict the resistance phenotype to rifampicin (RIF) and isoniazid (INH) in the additional independent MTB isolate datasets from India and Israel.

**Results:**

In our study, the Gradient Boosting Classifier (GBC) model was the best in terms of correctly identified percentages (97.28%, 96.06%, 94.19%, and 92.81% for the four first-line drugs, RIF, INH, pyrazinamide, and ethambutol respectively). By estimating the contributions of AMR-related SNPs by SHAP values, we found that position 761,155 (rpoB_p.Ser450), 2,155,168 (katG_p.Ser315) rank top in RIF and INH, their higher values (1 for alternative allele) tend to predict the resistance trait for these two drugs. In addition, the best model GBC generalizes well in predicting the resistance phenotypes for RIF and INH in the external independent MTB isolate datasets from India and Israel.

**Conclusions:**

This study integrates ML methods into antimicrobial resistance research, develops a framework for predicting resistance phenotypes, and explores AMR-related SNPs in MTB. Quantifying the important SNPs’ contribution to model decisions makes the ML algorithmic process more transparent, interpretable enabling and enables clinical practice.

**Supplementary Information:**

The online version contains supplementary material available at 10.1186/s12863-025-01338-x.

## Introduction

Tuberculosis (TB) is a human infectious disease caused primarily by *Mycobacterium tuberculosis* (MTB), and is the second leading cause of infectious disease deaths in the world [1]. An estimated 10.6 million new cases emerged globally in 2021, with 1.4 million fatalities attributed to TB [2]. Among these cases, rifampicin-resistant (RR) or multidrug-resistant (MDR) TB accounted for an estimated 3.6% of new infections, escalating to as high as 18% among previously treated patients [2]. Drug-resistant TB poses a significant challenge to global TB prevention and control efforts. Notably, China occupies the second position worldwide in reported cases of MDR-TB, trailing only India [3].

Traditional antibiotic susceptibility test (AST)-based methods take weeks to detect antimicrobial resistance (AMR) in MTB due to slow-growing. Current clinical diagnostic modalities, such as the Xpert MTB/RIF assay and linear probe assays (LPAs), are inherently limited in detecting resistance to a broader spectrum of antibiotics due to their focus on specific genomic targets [4, 5]. Whole Genome Sequencing (WGS) data, on the other hand, serves as a valuable instrument for monitoring genetic mutations in MTB, enabling early detection and preventive strategies against MDR-TB and extensively drug-resistant tuberculosis (XDR-TB) [6–8]. The World Health Organization’s (WHO) MTB mutation catalog proposes a panel of mutation sites for each common anti-tuberculosis drug resistance prediction, facilitating the provision of efficacious treatment options for individuals at risk of or diagnosed with drug-resistant TB [9].

Applying ML algorithms to integrated WGS data and AST phenotypes has facilitated the development of rapid predictive models for estimating drug resistance in MTB. This approach holds great promise for revolutionizing TB surveillance, diagnosis, and treatment. In recent years, ML algorithms such as support vector machine (SVM), logistic regression (LR), and random forest (RF) have been compared with variant-based association rules using WGS data from pathogen isolates for AMR prediction [10, 11]. ML has emerged as a powerful tool with higher specificity than risk-scoring methods [12]. For instance, Yang et al. assessed various ML models’ ability to categorize drug resistance for different MTB drugs and confirmed the utility of ML in identifying MTB resistance [10]. Kavvas et al. also developed a strain-agnostic computational platform that utilizes machine learning methods. This platform confirms 33 genes associated with resistance and discovers 24 new genetic signatures of AMR [13].

Despite the availability of commercial genotypic and phenotypic AST methods, they are insufficient for the comprehensive detection of drug-resistant (DR) profiles in patients diagnosed with TB. Secondly, the performance of WGS in predicting phenotypic drug resistance to anti-tuberculosis drugs is variable. Existing studies demonstrate that WGS yields highly concordant results (~ 90%) with AST phenotypes for first-line drugs, particularly isoniazid (INH) and rifampicin (RIF) [14, 15]. However, the predictive accuracy of WGS diminishes for ethambutol (EMB), pyrazinamide (PZA), and second-line anti-tuberculosis drugs [16, 17]. Consequently, it may be necessary to incorporate mutations beyond the WHO-recommended list to develop specific prediction models for resistance to these drugs. Notably, MTB isolates resistant to a single antibiotic may concurrently demonstrate susceptibility to alternative first- or second-line therapeutic options [4, 18]. In this study, we utilized WGS data from MTB isolates and their AST phenotypes against 18 antibiotics to construct a ML framework for predicting antimicrobial resistance. To enhance this technology’s eventual adoption and trustworthiness in public health settings, we also investigated the contributions of significant SNPs to the decision-making processes within our ML models. The source code and the training and validation datasets used in this study are available at GitHub: https://github.com/microbial123/MTB-AMR.

## Materials and methods

### *M. tuberculosis* isolates dataset and data pre-processing

We downloaded WGS data of 10,575 MTB isolates using prefetch as training data for ML models. Samples without AST phenotypes were filtered out. The data from the Sequence Read Archive were converted into FASTQ format using the fastq-dump tool. Based on the genomic quality assessment results of CheckM [19], we selected MTB samples with completeness > = 95% and contamination < 5%. Isolates with Q30 < 80% were filtered after quality control by using fastp [20]. Then sequencing reads were mapped to the reference genome MTB H37Rv strain (GenBank accession number NC_000962.3). SNP calling and genome annotation was performed using Snippy (v 4.6.0) [21]. Next, we used bcftools (v 1.20) [22] to merge SNP information from MTB isolates. Finally, 5739 MTB isolates with corresponding AST data were included in the subsequent analysis (Table S1).

The AST phenotypes of MTB to 18 antibiotics (amikacin (AMK), amoxicillin (AMX), capreomycin (CAP), ciprofloxacin (CIP), cycloserine (CYC), EMB, ethionamide (ETI), INH, kanamycin (KAN), moxifloxacin (MFX), nicotinamide, ofloxacin (OFX), para-aminosalicylic acid, prothionamide (PTH), PZA, rifabutin (RIB), RIF, and streptomycin (STR)) were obtained from the PATRIC database (http://www.patricbrc.org) as labels for model training [23].

Finally, 18 binary matrices were generated using python (v. 3.8.15) based on the genotypes (0 for SNPs without mutations and 1 for SNPs with mutations compared to the reference genome) of the MTBs and AST phenotypes (0 for susceptibility and 1 for resistance) for the 18 antibiotics. However, for ten datasets (including AMK, KAN, ETI, CAP, EMB, INH, MFX, OFX, PZA, RIF, and STR) with an abundance of SNP loci (> 30,000), feature selection was first conducted using the least absolute shrinkage and selection operator (LASSO) regression to mitigate computational load.

### Dataset

**Fig. 1:**
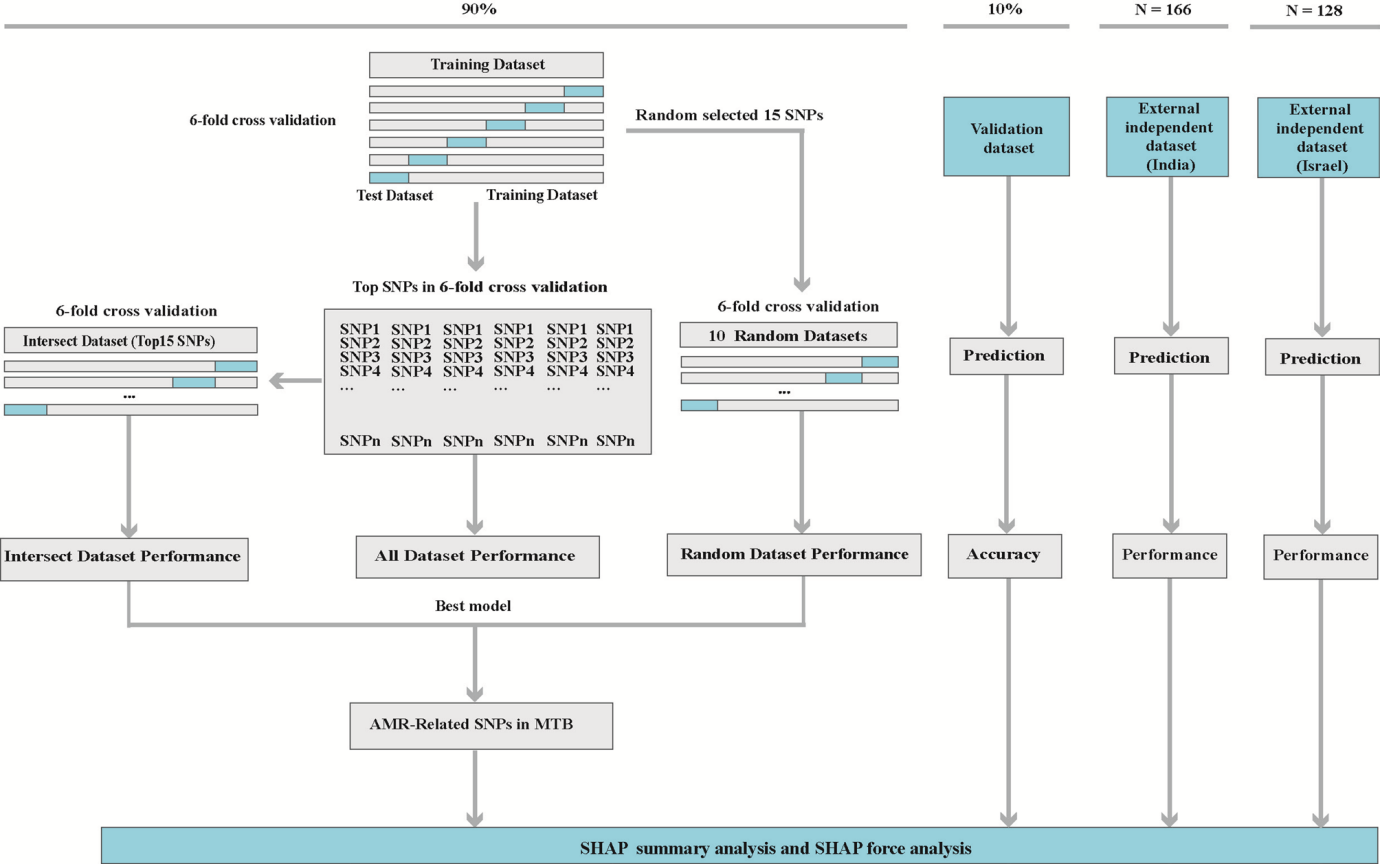
General workflow diagram illustrating the construction of optimal models for predicting resistance and identifying antimicrobial resistance-related SNPs in *Mycobacterium tuberculosis*. AMR, antimicrobial resistance; MTB, *Mycobacterium tuberculosis*; SNPs, single nucleotide polymorphisms; SHAP, SHapley Additive exPlanations

We randomly selected 10% of all isolates to be used as the validation dataset to ensure its independence from the model construction. The remaining 90% of the isolates were then used to construct three different datasets: the all, intersected, and random. All are SNPs included in the genotype dataset or selected by LASSO regression. Specifically, the ‘glmnet’ package in R was used to call the ‘cv.glmnet()’ function, which specifies a 10-fold cross-validation, to automatically calculate the cross-validation error for a series of λ values. We then used ‘lambda.min’ to train the final model and extracted the non-zero coefficients of the model as important features related to drug resistance. Intersected dataset: We listed the AMR genes that ranked in the top 15 in terms of importance in predicting resistance phenotypes by the optimal model in an n-fold (*n* = 5, 6) cross-validation setting. It was used to train the machine algorithm and reevaluate its performance. Random dataset: We randomly selected 15 SNPs from all datasets and performed 10 replications, eventually generating 10 “random datasets” (Fig. 1).

### Machine learning algorithms

Algorithm Selection: We employed twelve ML algorithms: Logistic Regression (logR), Gaussian Naive Bayes (gNB), Support Vector Machine (SVM), Decision Trees (DT), Random Forest (RF), K-Nearest Neighbors (KNN), Linear Discriminant Analysis (LDA), Multinomial Naive Bayes (mNB), AdaBoost Classifier (ABC), Gradient Boosting Classifier (GBC), Extra Trees Classifier (ETC), and Bagging Classifier (BC).

We constructed ML models using 90% of the collected MTB isolates and obtained a series of drug resistance-related SNPs according to their importance in 6-fold cross-validations. Based on the key resistance-related SNPs screened out in the training set above, the performance and stability of the model were evaluated using the remaining fold of the test dataset. The model was then applied to the entire dataset and predicted in an external independent validation dataset to assess its predictive accuracy.

### Performance evaluation

Precision, recall, F1-score, the area under the receiver operating characteristic curve (au ROC), the area under the precision-recall curve (au PR), and 10-fold cross-validated classification accuracy metrics are computed by Scikit-learn (v1.2.2) to assess the performance of the twelve models across the three datasets. A flowchart outlining the process of constructing a model for predicting drug resistance phenotype and identifying resistance-related genes in MTB is shown in Fig. 1.

### Annotation and functional analysis of AMR-related SNPs

The ML framework identified a set of potential AMR-related SNPs in MTB. To elucidate AMR-associated genes, we performed genomic annotation of these SNPs using SnpEff (v2.0) [24].

### Interpretability for the ML model

We extracted the SNPs in the GBC model using python and plotted the importance scores according to their rankings. To understand why and how decisions were made in the selected optimal ML algorithm, we then used a SHAP summary plot to illustrate the effects of each SNP attributed to the model [25]. After selecting the best-performing model based on prediction accuracy in each MTB-antibiotic group, we used the model to predict the resistance/susceptible phenotype for the specific drugs in randomly selected MTB isolates from the external independent dataset. Next, SHAP force plots were used to visualize the impact of important SNPs on the prediction results in the single isolate. All analyses were performed using Python (v3.8.15).

### Independent dataset validation

We used two independent datasets for validation to evaluate the generalization ability of ML models trained on the genotypes and drug-resistant phenotypes of MTB isolates. These datasets consisted of 166 multidrug-resistant MTB isolates collected from patients in Chennai, India (NCBI Bioproject accession ID: PRJNA741102) and 128 MTB isolates from Israel (NCBI Bioproject accession ID: PRJNA957554). We downloaded the SRA data and performed quality control, genome mapping, and SNP calling through the process of data processing described above (see Materials and Methods, *M. tuberculosis isolates* dataset and Data Pre-processing), which resulted in a genotype matrix containing all SNPs for all isolates. Finally, we used the GBC model trained with the intersected SNPs (top15 SNPs across 6-fold) to predict their resistance/susceptible traits. The results of the predictions were compared to those of the drug susceptibility tests provided in the original article [8, 26], and the performance of the machine learning models was completed in Python (v3.8.15).

GenTB is a well-established user-friendly whole genome-based predictor for tuberculosis resistance powered by machine learning [27]. Both our machine learning framework and GenTB were applied to predict the drug-resistance phenotypes of the MTB isolates in the two independent datasets. Key performance metrics, including accuracy, precision, recall, and F1-score, were calculated for each prediction method. These metrics were utilized to assess the efficacy of our machine learning framework and GenTB in predicting resistance to tuberculosis.

## Results

**Fig. 2:**
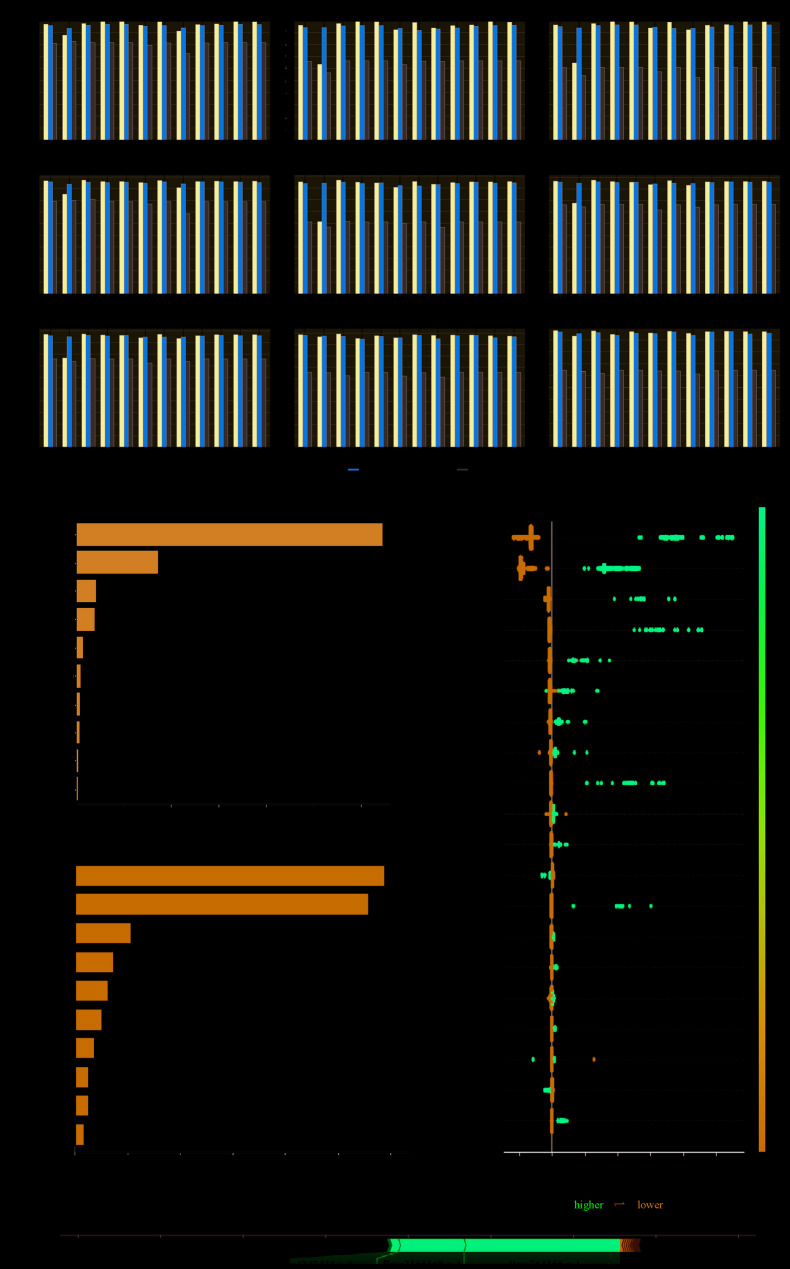
Assessment of the performance of the machine learning algorithms in predicting resistance to rifampicin by MTB in 6-fold cross validation settings and interpretability for the ML model. **A** The preformance metrics. (i) training precision, (ii) training recall, (iii) training F1, (iv) test precision, (v) test recall, (vi) 10-fold CV (cross validation), (vii) Loo CV (leave-one-out cross validation), (viii) au ROC (area under ROC curve) and (ix) au PR (area under precision recall curve). ‘All’ denotes all SNPs for training (as in the cross-validation partitioning), ‘Intersection’ refers to AMR SNPs that consistently ranked high across all 6 rounds of cross-validation, and ‘Random’ refers to randomly sampled SNPs. **B** The interpretability for the ML model. (i) The importance of each SNP in building the final predictive model in RIF. (ii) SHAP summary plot of SNPs contributing to the GBC model in RIF. X-axis shows the average of the absolute SHAP values. (iii) SHAP values of SNPs in the GBC model. Y-axis lists the different SNPs, and X-axis shows the SHAP values. Each dot in the plot represents an MTB isolate. The color of the dots from blue to red shows the feature values from low to high. (iv) SHAP force plot for explaining of a single MTB isolate’s prediction result. SHAP, SHapley Additive exPlanations; GBC, Gradient Boosting Classifier

This study presents a ML framework based on 12 algorithmsapplied to many MTB isolates to predict their susceptibility/resistance phenotypes to 18 antibiotics. We found that no single model consistently performed well in all cases. Thus, we summarize the top three best models (based on F1-score) for each MTB-drug group (Table S3). Notably, DT, ETC, and LogR outperformed others in the training set for all datasets. Conversely, GBC, LogR, and SVM emerged as the top three classifiers in the test set. As for the intersected datasets, the top three classifiers in the training set were DT, SVM, and ETC, while in the test set, SVM, LDA, and ABC exhibited superior performance. The performances of 12 classifiers in predicting MTB resistance to 18 antibiotics are shown in Fig. 2 and Figure S2A-S18A. The performance evaluation of each ML model encompassed precision, recall, F1-score, area under the Receiver Operating Characteristic curve (au ROC), and area under the Precision-Recall curve (au PR). The performances of 12 ML algorithms in predicting antimicrobial resistance to different drugs are depicted in Fig. 2A and Figure S2A-S18A. The F1-score for the intersected dataset are comparable to better than those for all datasets and higher than for the random dataset. In addition, the performances of ML models with 5-fold cross-validation decreased compared to those with 6-fold.

Table S4 provides a comprehensive comparison of the predicted and actual labels of the 12 ML models on an independent prediction dataset that was not involved in feature selection and model training. In assessing the prediction accuracy for 18 antibiotics, the top three classifiers with the highest frequency are GBC, BC, and ABC. Especially remarkable was the GBC classifier’s capability to accurately predict the resistance traits of RIF in MTB isolates, with an outstanding correctness rate of approximately 97.28%. In contrast, other methods, such as gNB, LogR, and SVM, have lower or comparable accuracy rates, ranging from 74.48% for gNB, 96.44% for LogR, to 97.07% for SVM (Table S4).

To understand why and how decisions were made in the optimal ML algorithm, we extracted the AMR-related SNPs in the GBC model and showed the importance according to their rankings (Fig. 2B i and Figure S2B-S18B i). Figure 2B ii) and iii) depict the SHAP summary plot of SNPs contributing to the GBC model in RIF. The top three significant variants in RIF were located at the position 761155 (rpoB_p.Ser450), 2155168 (katG_p.Ser315), 761110 (rpoB_p.Asp435) in NC_000962.3. The SHAP values of these SNPs were positive, which means that their higher values in the MTB genomic matrix (1 indicates alternative allele) tend to predict resistance traits in RIF (Fig. 2B iv). While the top three significant variants in INH were located at the position 2155168 (katG_p.Ser315), 761155 (rpoB_p.Ser450), 1673425 (inhA_c.−777 C), see Figure S2B ii and iii. The SHAP summary plots and SHAP force plots showed the impact of important SNPs on the prediction results of 18 different drugs in the MTB isolates (Fig. 2B iv and Figure S2iv-S18 iv).

Table 1 lists the AMR variants our ML models discerned alongside their respective incidences across 18 distinct drugs. Remarkably, of the initial fifteen variants enumerated, fourteen find mention within the WHO’s Catalogue of mutations in MTB Complex (2nd edition), with twelve of these mutations specifically tied to resistance against at least one antibiotic, classified under the designations “associated with resistance” (Ass-w-R) or “associated with resistance-intermediate” (Ass-w-R-int) [9]. See Table S5 for details. Table S5 lists the details of the potential resistance-associated SNPs in each antibiotic identified by the ML model.

**Table 1:** A list of potential resistance-associated SNPs in MTB that consistently appeared among the most important features targeted in each round of 6-fold cross-validation for each of the 18 antibiotics

Index	Chromosome	Position	Variant	REF	ALT	Gene	Amikacin	Amoxicillin	Capreomycin	Ciprofloxacin	Cycloserine	Ethambutol	Ethionamide	Isoniazid	Kanamycin	Moxifloxacin	Nicotinamide	Ofloxacin	Para-aminosalisylic_acid	Prothionamide	Pyrazinamide	Rifabutin	Rifampicin	Streptomycin	Frequence
1	NC_000962.3	1473246	rrs_n.1401A>G	A	G	*rrs*	√		√		√	√	√		√		√	√			√		√	√	11
2	NC_000962.3	7570	gyrA_p.Ala90Glu; gyrA_p.Ala90Gly; gyrA_p.Ala90Val; gyrA_p.Ser91Pro	C/CGT	T,TGC,G	*gyrA*			√	√		√	√		√	√	√	√			√	√		√	11
3	NC_000962.3	2155168	katG_c.942C>A; katG_p.Ser315Asn; katG_p.Ser315Ile; katG_p.Ser315Thr	C/CTGGT	G,T,A,GTGGC	*katG*			√			√		√		√		√			√	√	√	√	9
4	NC_000962.3	761110	rpoB_p.Asp435Ala; rpoB_p.Asp435Gly; rpoB_p.Asp435Val	A	T,G	*rpoB*				√		√		√	√		√			√			√	√	8
5	NC_000962.3	761155	rpoB_c.1353G>C; rpoB_c.1356G>T; rpoB_c.1359G>T; rpoB_p.Ala451Gly; rpoB_p.Ser450Cys; rpoB_p.Ser450Leu; rpoB_p.Ser450Phe; rpoB_p.Ser450Trp; rpoB_p.Ser450Tyr	C/CG	G,T,TC,TT	*rpoB*						√		√	√	√					√	√	√	√	8
6	NC_000962.3	4247429	embB_p.Met306Leu; embB_p.Met306Val	A	C,G	*embB*						√	√	√							√	√	√	√	7
7	NC_000962.3	7582	gyrA_p.Asp94Ala; gyrA_p.Asp94Gly; gyrA_p.Asp94Val; gyrA_p.Ser95Ala; gyrA_p.Ser95Thr	ACAG/A	GCAC,CCAC,TCAC,C,G	*gyrA*				√	√	√			√	√		√							6
8	NC_000962.3	7585	gyrA_c.286C>T; gyrA_c.288G>C; gyrA_c.291G>C; gyrA_c.294C>G; gyrA_p.Ser95Thr	G/GCCTG	C,CCCTA	*gyrA*	√			√			√		√	√		√							6
9	NC_000962.3	1673432	inhA_c.-770T>A; inhA_c.-770T>C; inhA_c.-770T>G	T	A,G,C	*inhA*			√			√			√	√	√								5
10	NC_000962.3	4247431	embB_c.921C>G; embB_c.924A>G; embB_c.927C>G; embB_p.Met306Ile	G,GGCCCGAGTC	C,A,T,CGCGCGGGTG	*embB*				√		√									√	√	√		5
11	NC_000962.3	4269271	ubiA_p.Val188Ala; ubiA_p.Val188Gly	A	C,G	*ubiA*				√					√		√	√							4
12	NC_000962.3	761161	rpoB_p.Leu452Pro	T	C	*rpoB*							√	√	√						√				4
13	NC_000962.3	763123	rpoB_p.Ile1106Thr	T	C	*rpoB*				√					√	√	√								4
14	NC_000962.3	1218896		G	A		√		√												√				3
15	NC_000962.3	1673425	inhA_c.-777C>T	C	T	*inhA*							√	√										√	3

*MTB* Mycobacterium tuberculosis, *REF* Reference, *ALT* alternative

Through genetic annotation of potential MTB resistance SNPs, we identified numerous previously reported genes, including DNA gyrase subunit A (*gyrA*), DNA-directed RNA polymerase subunit beta (*rpoB*), putative arabinosyltransferase B (*embB*), and Catalase-peroxidase (*katG*), see Table 1 and Figure S19 for details. In addition, we identified a list of potential resistance-associated genes that have not been mentioned in the WHO’s catalog, such as putative PE-PGRS family protein PE_PGRS54 (in CYC, ETI, and KAN), and multidrug efflux system permease protein (in EMB, OFX, RIF, and STR), cation-transporter P-type ATPase B (in nicotinamide, RIF, and STR), ATP-dependent zinc metalloprotease FtsH (in CIP, KAN, nicotinamide), and putative arabinosyltransferase A (in EMB, ETI and STR), etc. These SNPs are predominantly associated with the functions of transporter proteins and transferase enzymes on the cell membrane, playing vital roles in bacterial metabolic processes.

**Fig. 3:**
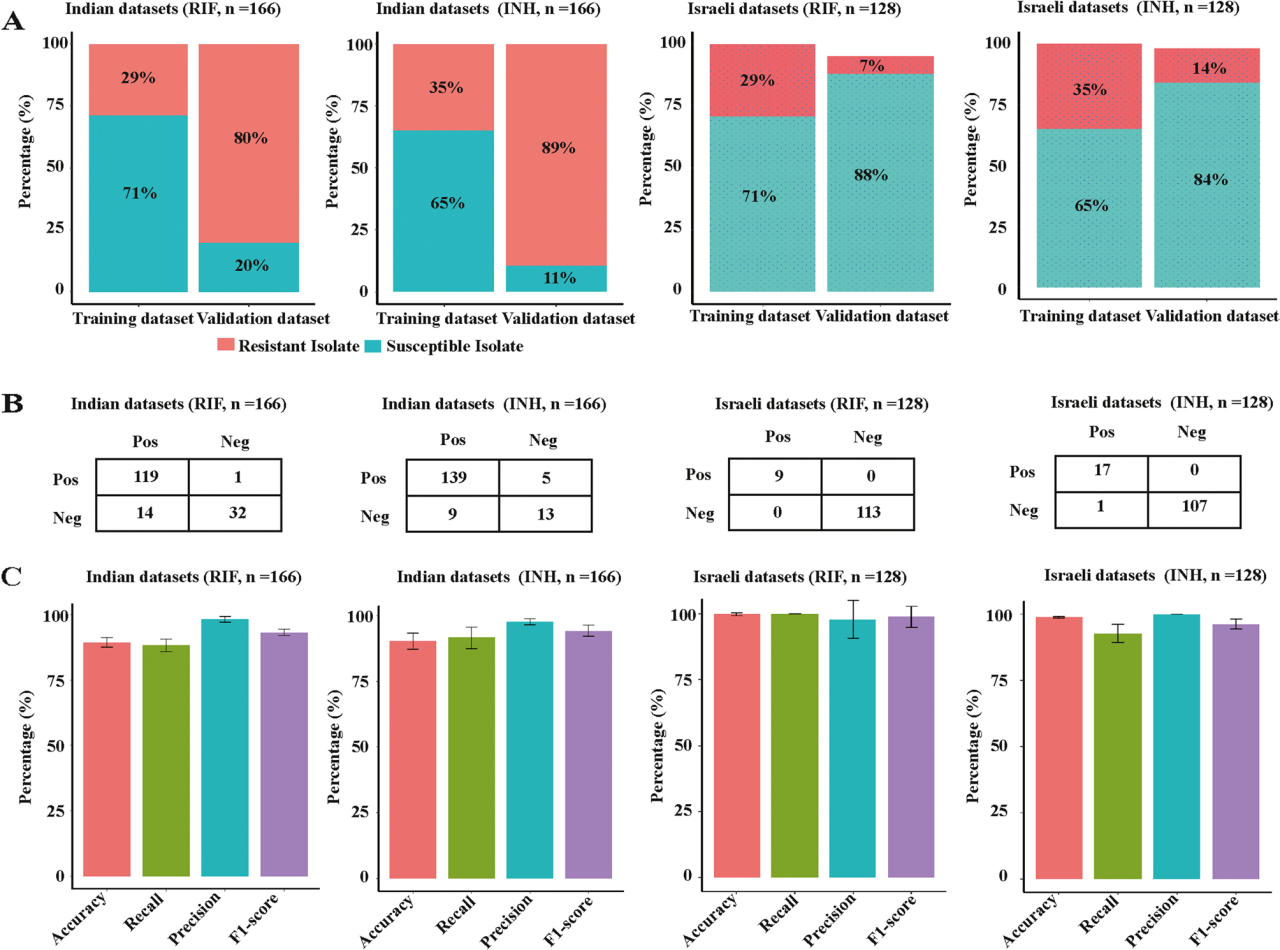
Application of Machine Learning Models for Drug Resistance Phenotype Prediction in an Independent Dataset. **A** The proportion of resistant and susceptible isolates of MTB in training datasets and external independent validation dataset. The sum of the proportions of strains in the graph is not 100% due to the presence of “NA” (missing values) and “I” (intermediate) in the datasets. **B** Prediction results of the machine learning algorithms in predicting resistance phenotype to rifampicin and isoniazid. **C** The performance of 12 machine learning models on the external validation datasets

In the independent Indian dataset, resistance rates to RIF, INH, EMB, and PZA were 80%, 89%, 13%, and 36%, respectively (Figure S20A). Lineage 1 accounted for 50% of the isolates (Figure S20B). Conversely, the Israeli dataset exhibited resistance rates of 7%, 14%, 3%, and 9% to the four antibiotics (Figure S20A). Lineage 4 was the most prevalent, constituting 41.88% of the isolates (Figure S20B). In the external verification datasets, the ML models trained on MTB genotypic and phenotypic data accurately predicted the resistance/susceptible phenotypes for RIF and INH. The performances of the models are shown in Fig. 3A-C. The GBC algorithm correctly predicted the resistance trait to RIF and INH over 90% of MTB isolates (Fig. 3C). However, for EMB and PZA, the average accuracy was 83.73% (India, EMB), 67.42% (India, PZA), 99.05% (Israel, EMB), and 91.14% (Israel, PZA), respectively (Table S6). The models are challenging to generalize well in the Indian verification dataset.

In addition, we used the same two independent datasets for our machine learning framework and GenTB, comparing key performance metrics such as accuracy, precision, recall and F1-score in predicting tuberculosis resistance. For RIF prediction, the GBC model achieved F1-score of 0.941 (India) and 1.000 (Israel), while GenTB achieved 0.946 (India) and 1.000 (Israel). In INH prediction, the GBC model outperformed GenTB in India (0.952 vs. 0.935) and Israel (0.971 vs. 0.829). Notably, the GBC model demonstrated superior performance for EMB in India (0.510 vs. 0.474) and Israel (1.000 vs. 0.889). However, both models performed poorly for PZA, with the GBC model scoring 0.290 (India) and 0.267 (Israel), compared to GenTB’s 0.333 (India) and 0.183 (Israel). The comparison has been presented in Table S7.

## Discussion

The combination of WGS and ML is promising as an advanced technology for accurately predicting different anti-TB drug resistance. Previous ML models typically focused on common anti-tuberculosis drugs, our study constructed prediction models not only for the four first-line drugs (INH, RIF, EMB, PZA) but also for fourteen second-line drugs (AMK, AMX, CIP, CYC, CAP, ETI, KAN, MFX, nicotinamide, OFX, para-aminosalicylic acid, PTH, RIB, STR), incorporating mutation sites beyond the WHO-recommended list. This approach enabled us to predict drug resistance traits for specific antibiotics upon acquiring WGS data, addressing the limitations of current WHO mutation catalogs [9], Xpert MTB/RIF assay, and LPAs [4, 5]. This framework deepens our understanding of the genetic basis of AMR, and quantifying the critical SNPs’ contribution to model decisions makes the ML algorithmic process more interpretable, and enables clinical practice.

In this study, we selected SNPs from WGS data for ML models training, extending beyond the limited mutation catalog recommended by the WHO [9]. LASSO-screened features show strong performance in training and test datasets for all 12 algorithms. The top three classifiers (based on F1-score) were Linear LDA, GBC, and SVM, respectively. When applied to an external independent MTB dataset, GBC was the best performing of the 12 models, demonstrating the accuracy of 92.81%, 96.06%, 94.19%, and 97.28% in predicting resistance to EMB, INH, PZA, and RIF. GBC classifier uses an iterative learning strategy to sequentially refine the model by focusing on the residuals of misclassified instances. This incremental optimization process enhances the capabilities of the model. Furthermore, the models showed good generalizability on an independent dataset of MTB isolates from India [8]. However, the predictions for PZA and EMB were less satisfactory (Table S6), echoing findings from prior research conducted in China [15, 16], Thailand [17], India [8], Indonesia [28], and a study based on WHO-recommended MTB mutation sites [7]. A Middle Eastern study reported perfect prediction rates for EMB resistance using WGS data [29]. These drug discrepancies may be attributed to genetic variations among MTB isolates in different regions. Regarding the generalizability of prediction models for other second-line drugs, we did not validate them on independent datasets in the present study. This will be examined in future research to improve our understanding and predictive abilities.

In a 6-fold cross-validation of the dataset, consistently top-ranked genes (e.g., *gyrA*, *rpoB*, *embB*, *inhA*, and *katG*) were associated with antimicrobial resistance in MTB. Of particular interest, the positions 1473246 and 7570 emerge as SNPs of resistance across eleven drugs. The WHO catalog emphasizes the mutation rrs_n.1401 A > G at position 1,473,246, constituting Group 1 of resistance to AMK, KAN, and CAP (Assoc-w-R). At position 7570, two mutational variants, gyrA_p.Ala90Val and gyrA_p.Ser91Pro was identified, exhibiting a significant correlation with Levofloxacin resistance and thus categorized as Ass-w-R in the catalog. Additionally, position 761155 emerged as the foremost determinant of RIF resistance in our assessments. This variant was classified as Ass-w-R within the WHO catalog [9], aligning with the oft-cited *rpoB*_S450L mutation in the Asian context [30]. Located within the Rifampicin Resistance Determining Region (RRDR), the substitution of serine (S) by leucine (L) at codon 450 has been implicated in heightened resistance against RIF [31]. The *rpoB* gene, which encodes for RNA polymerase B, has almost 95% of the mutations clustered in the codon region known as the RIF resistance determination region [32, 33]. The *katG*-encoded catalase KatG activates isoniazid [34], leading to its adduct formation with NAD and inhibition of the NADH-dependent enoyl-carrier protein reductase InhA, which in turn inhibits the biosynthesis of mycophenolic acid [35, 36]. The mutations of *katG* and up-regulation or targeted modification of InhA in MTB can lead to resistance to isoniazid [34]. Our annotation ofposition 1473246 by SnpEff suggests an upstream variant, ISL3 family transposase IS1557. Insertion sequences (IS) are removable DNA fragments that play a key role in transmitting resistance genes by inserting them into new sites within or between the cell’s DNA strands [37]. A previous report indicated that a highly rifampicin-resistant strain of MTB from southern Brazil had an uncommon 12-nucleotide insertion at codon 435 of the *rpoB* [38]. Rudy Antoine et al. showed that IS6110-mediated gene disruption is a clinically relevant mechanism for developing antibiotic resistance in MTB and should be considered for molecular diagnostics [39].

We compared the performance of our best model (GBC) with GenTB in Indian and Israeli cohorts. The GBC model demonstrated overall stronger performance compared to GenTB, particularly in accuracy and precision for EMB and PZA across both cohorts. The GBC model and GenTB exhibited comparable performance in predicting RIF and INH resistance. Remarkably, the GBC model matched the predictive accuracy of GenTB’s whole-genome analysis using just 15 SNPs, demonstrating the critical importance of these key genetic markers. Notably, the stark performance gap for PZA (e.g., GBC’s 0.290 F1-score vs. GenTB’s 0.333 in India) underscores the challenges in predicting resistance for this drug, likely due to limited biomarker specificity. Future research should explore hybrid models integrating SNP-focused precision with WGS breadth, alongside efforts to refine PZA-specific biomarkers for both platforms.

While the ML framework successfully identified genetic mutation loci associated with MTB resistance, our study has several limitations. Firstly, there is a notable imbalance in the phenotypes of resistant/susceptible across different antibiotic datasets for MTB, with a considerably higher number of susceptible isolates in most datasets. This imbalance could potentially limit the performance and generalizability of certain analyses. However, future research may need to consider techniques such as oversampling the resistant isolates or using more complex ML algorithms robust to class imbalance. This will help ensure that the results better represent the true distribution of drug-resistant and susceptible MTB in the broader population. Secondly, our study focused solely on resistance mutations at the SNP and gene levels, lacking a comprehensive understanding of the overall genome-scale model of MTB. Further research is imperative to elucidate and comprehend antimicrobial resistance’s genetic and epigenetic features from a genome-wide and multi-omics perspective. Thirdly, although our ML model indicated a potential correlation between top SNPs and anti-tuberculosis resistance phenotype, it did not establish a causal relationship between SNPs and resistance phenotypes. The ML algorithm did not predefine the mechanisms of drug resistance before analysis. Instead, it was based on SNPs at the whole-genome level, with no consideration of structural and functional linkages. Additionally, future work should focus on collections with more phenotypic resistance to new and repurposed drugs, which could involve in vivo and in vitro selection experiments.

Several studies have explored ML models for predicting MTB resistance phenotypes [13, 40]. Although these studies claimed that some algorithms performed well in prediction accuracy, the non-interpretability of the black box hindered the practice and extended application of these predictive models in the clinical setting [41]. By using the SHAP summary plot, we found the top 3 variants located at the position 761,155 (rpoB_p.Ser450), 2,155,168 (katG_p.Ser315), 761,110 (rpoB_p.Asp435) have positive SHAP values. Their higher values in the MTB genome matrix (1 for alternative allele) tend to the prediction of resistance for RIF. Quantifying these SNPs’ contribution to model decisions makes the ML algorithmic process more transparent and interpretable and enables clinical practice. We should follow privacy protection measures when applying ML models to process AMR data (including data acquisition, storage, and analysis). Notably, in clinical implementation, the accuracy of AMR prediction is directly related to patient treatment options and health outcomes. The false-positive and false-negative results may increase the risks of overtreatment, antibiotic misuse, treatment failure, etc. Therefore, we need to enhance their integration with clinical judgment further to reduce the negative impact of these errors and improve the reliability of clinical decisions.

## Conclusions

This research underscores the significance of integrating ML methodologies into the study of antimicrobial resistance. In summary, we integrated WGS data and the AST phenotype of MTB, utilizing ML to establish a framework for antimicrobial resistance and susceptible SNPs in MTB. This framework enhanced our comprehension of the genomic genetic variability in MTBs. The predictive capacity holds promise for guiding future clinical decisions regarding antibiotic use in treating MTB-related diseases. Looking forward, enhanced machine learning frameworks could address the ongoing antibiotic resistance crisis and effectively treat diseases caused by emerging pathogens that significantly threaten human health.

## Supplementary Information

Supplementary Material 1: Figure S1. Proportion of drug-resistant and susceptible isolates of Mycobacterium tuberculosis in the datasets used in this study. The blue group represents drug-susceptible isolates, and the red group represents drug-resistant isolates. Figure S2-S18. Assessment of the performance of the machine learning algorithms in predicting resistance to 17 antibiotics by MTB in 6-fold cross validation settings and interpretability for the ML model. (A) The preformance metrics. (i) training precision, (ii) training recall, (iii) training F1, (iv) test precision, (v) test recall, (vi) 10-fold CV (cross validation), (vii) Loo CV (leave-one-out cross validation), (viii) au ROC (area under ROC curve) and ix) au PR (area under precision recall curve). ‘All’ denotes all SNPs for training (as in the cross-validation partitioning), ‘Intersection’ refers to AMR SNPs that consistently ranked high across all 6 rounds of cross-validation, and ‘Random’ refers to randomly sampled SNPs. (B) The interpretability for the ML models. (i) The importance of each SNP in building the final predictive model. (ii) SHAP summary plot of SNPs contributing to the GBC model. X-axis shows the average of the absolute SHAP values. (iii) SHAP values of SNPs in the GBC model. Y-axis lists the different SNPs, and X-axis shows the SHAP values. Each dot in the plot represents an MTB isolate. The color of the dots from blue to red shows the feature values from low to high. (iv) SHAP force plot for explaining of a single MTB isolate’s prediction result. SHAP, SHapley Additive exPlanations; GBC, Gradient Boosting Classifier. Figure S19. The potential common genes for MTB resistance to different antibiotics. The ribbons connecting drugs to mutation genes represent the associations. The longer the length of the outer ring, the more antibiotics (genes) are associated with the gene (antibiotic). Figure S20. Proportion of drug-resistant and susceptible isolates of MTB in two independant datasets. (A) MTB isolates collected from Chennai, India (NCBI Bioproject accession ID: PRJNA741102) and MTB isolates from Israel (NCBI Bioproject accession ID: PRJNA957554). Note: R, resistant. S, susceptible. I, intermidiate. NA, not available. (B) Proportions of Different Lineages in Two Independent MTB Datasets.

Supplementary Material 2: Table S1. Publicly available whole genome sequencing datasets used in this study. Table S2. The number of isolates susceptible or resistant to antibiotics and the total number of SNPs in each group used for training and testing of antibiotic-specific machine learning models. Table S3. Identification of the top three best models (based on F1-score) for each MTB-drug group. Table S4. Percentage of correctly identified resistance phenotype to the specific drug in an independent dataset of MTB isolates by 12 machine learning algorithms. Table S5. The details of potential resistance-related SNPs in each antibiotic. Table S6. Assessment of the performance of the optimal machine learning algorithms in predicting resistance to four first-line drugs in the external independent MTB isolates. Table S7. Comparative Evaluation of MTB Isolate Resistance Phenotype Prediction between Our Machine Learning Framework and GenTB Using two Independent Datasets.

## Data Availability

WGS data for 5,739 MTB isolates were downloaded from the Sequence Read Archive (SRA) database and the corresponding antibiotic susceptibility test (AST) phenotypes to 18 antibiotics were obtained from the PATRIC database (http://www.patricbrc.org). The individual accession numbers are listed in Table S1. The external independent validation dataset used in this study (containing genomic and resistance phenotype data for 166 MTB isolates) was collected from patients in Chennai, India. (NCBI Bioproject accession ID: PRJNA741102). The external independent validation dataset used in this study (containing genomic and resistance phenotype data for 128MTB isolates) was collected from patients in Israeli. (NCBI Bioproject accession ID: PRJNA957554).

## Abbreviations

ABC: Adaboost Classifier
AMK: Amikacin
AMR: Antimicrobial Resistance
AMX: Amoxicillin
au PR: Area under the precision-recall curve
au ROC: Area under the receiver operating characteristic curve
Ass-w-R: Associated with Resistance
Ass-w-R-int: Associated with Resistance-Intermediate
AST: Antibiotic Susceptibility Test
BC: Bagging Classifier
CAP: Capreomycin
CIP: Ciprofloxacin
CYC: Cycloserine
DR: Drug-Resistant
DT: Decision Trees
EMB: Ethambutol
embB: Putative Arabinosyltransferase B
ETC: Extra Trees Classifier
ETI: Ethionamide
GBC: Gradient Boosting Classifier
gNB: Gaussian Naive Bayes
gyrA: Dna Gyrase Subunit A
INH: Isoniazid
IS: Insertion Sequences
KAN: Kanamycin
katG: Catalase-Peroxidase
KNN: K-Nearest Neighbors
LASSO: Least Absolute Shrinkage and Selection Operator
LDA: Linear Discriminant Analysis
logR: Logistic Regression
LPAs: Linear Probe Assays
LR: Logistic Regression
MDR: Multidrug-Resistant
MFX: Moxifloxacin
ML: Machine Learning
mNB: Multinomial Naive Bayes
MTB: Mycobacterium Tuberculosis
OFX: Ofloxacin
PTH: Prothionamide
PZA: Pyrazinamide
RF: Random Forest
RIB: Rifabutin
RIF: Rifampicin
rpoB: DNA-Directed RNA Polymerase Subunit Beta
RR: Rifampicin-Resistant
RRDR: Resistance Determining Region
SHAP: Shapley Additive Explanations
SNP: Single Nucleotide Polymorphism
SRA: Sequence Read Archive
STR: Streptomycin
SVM: Support Vector Machine
TB: Tuberculosis
WGS: Whole Genome Sequencing
WHO: World Health Organization
