# Supplementary material for: Machine learning-based prediction of antimicrobial resistance and identification of AMR-related SNPs in Mycobacterium tuberculosis
Source: BMC Genom Data. 2025 Jul 12;26:48. doi: 10.1186/s12863-025-01338-x (PMC12255030; doi:10.1186/s12863-025-01338-x)
Supplement: Supplementary file 1 — Supplementary Material 1: Figure S1. Proportion of drug-resistant and susceptible isolates of Mycobacterium tuberculosis in the datasets used in this study. The blue group represents drug-susceptible isolates, and the red group represents drug-resistant isolates. Figure S2-S18. Assessment of the performance of the machine learning algorithms in predicting resistance to 17 antibiotics by MTB in 6-fold cross validation settings and interpretability for the ML model. (A) The preformance metrics. (i) training precision, (ii) training recall, (iii) training F1, (iv) test precision, (v) test recall, (vi) 10-fold CV (cross validation), (vii) Loo CV (leave-one-out cross validation), (viii) au ROC (area under ROC curve) and ix) au PR (area under precision recall curve). ‘All’ denotes all SNPs for training (as in the cross-validation partitioning), ‘Intersection’ refers to AMR SNPs that consistently ranked high across all 6 rounds of cross-validation, and ‘Random’ refers to randomly sampled SNPs. (B) The interpretability for the ML models. (i) The importance of each SNP in building the final predictive model. (ii) SHAP summary plot of SNPs contributing to the GBC model. X-axis shows the average of the absolute SHAP values. (iii) SHAP values of SNPs in the GBC model. Y-axis lists the different SNPs, and X-axis shows the SHAP values. Each dot in the plot represents an MTB isolate. The color of the dots from blue to red shows the feature values from low to high. (iv) SHAP force plot for explaining of a single MTB isolate’s prediction result. SHAP, SHapley Additive exPlanations; GBC, Gradient Boosting Classifier. Figure S19. The potential common genes for MTB resistance to different antibiotics. The ribbons connecting drugs to mutation genes represent the associations. The longer the length of the outer ring, the more antibiotics (genes) are associated with the gene (antibiotic). Figure S20. Proportion of drug-resistant and susceptible isolates of MTB in two independant datasets. [file 12863_2025_1338_MOESM1_ESM.pdf]

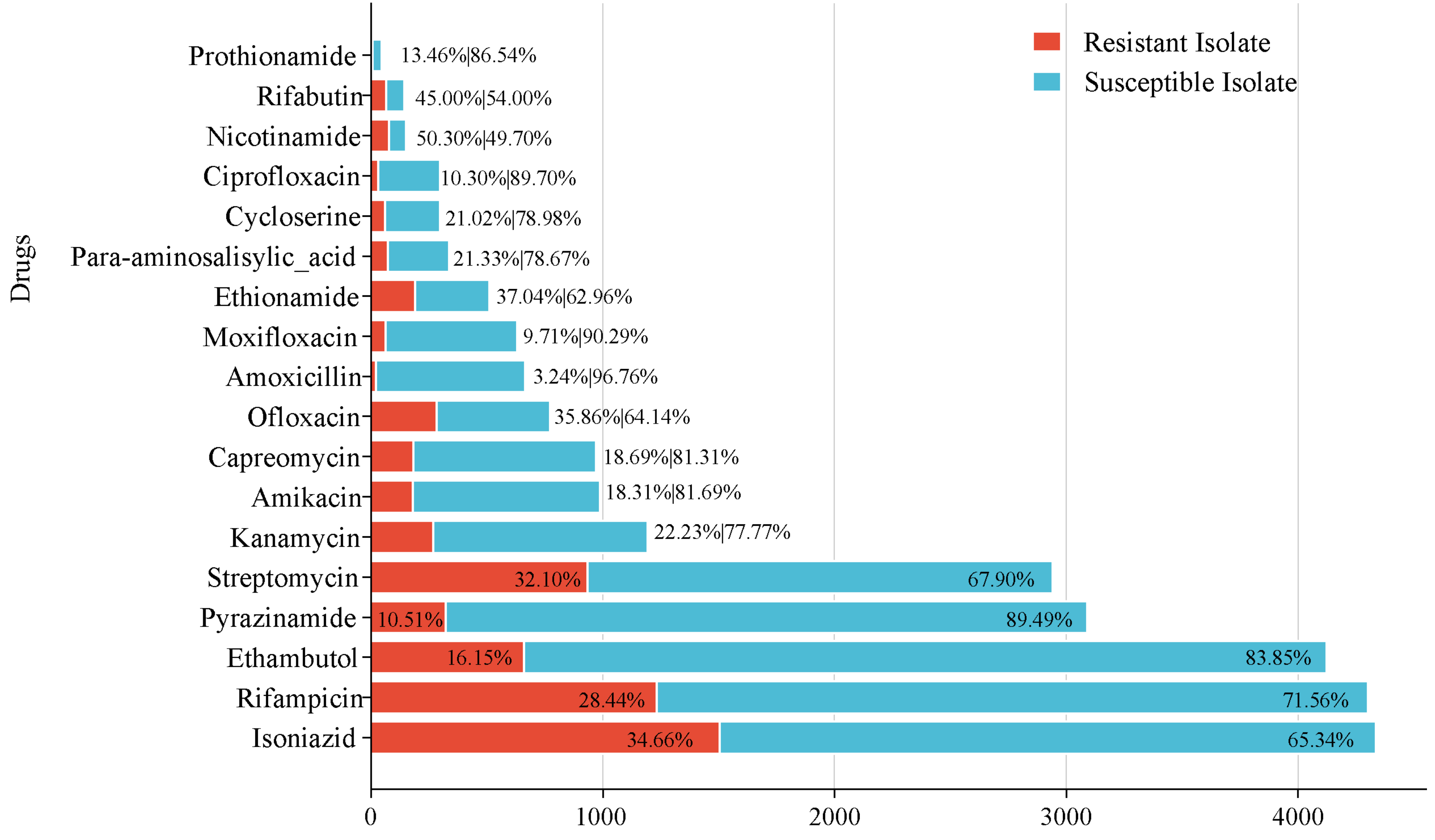
Resistant Isolate
Susceptible Isolate
Drugs
Prothionamide
Rifabutin
Nicotinamide
Ciprofloxacin
Cycloserine
Para-aminosalisylic_acid
Ethionamide
Moxifloxacin
Amoxicillin
Ofloxacin
Capreomycin
Amikacin
Kanamycin
Streptomycin
Pyrazinamide
Ethambutol
Rifampicin
Isoniazid
13.46%|86.54%
45.00%|54.00%
50.30%|49.70%
10.30%|89.70%
21.02%|78.98%
21.33%|78.67%
37.04%|62.96%
9.71%|90.29%
3.24%|96.76%
35.86%|64.14%
18.69%|81.31%
18.31%|81.69%
22.23%|77.77%
32.10%
67.90%
10.51%
89.49%
16.15%
83.85%
28.44%
71.56%
34.66%
65.34%
0
1000
2000
3000
4000

Figure S1

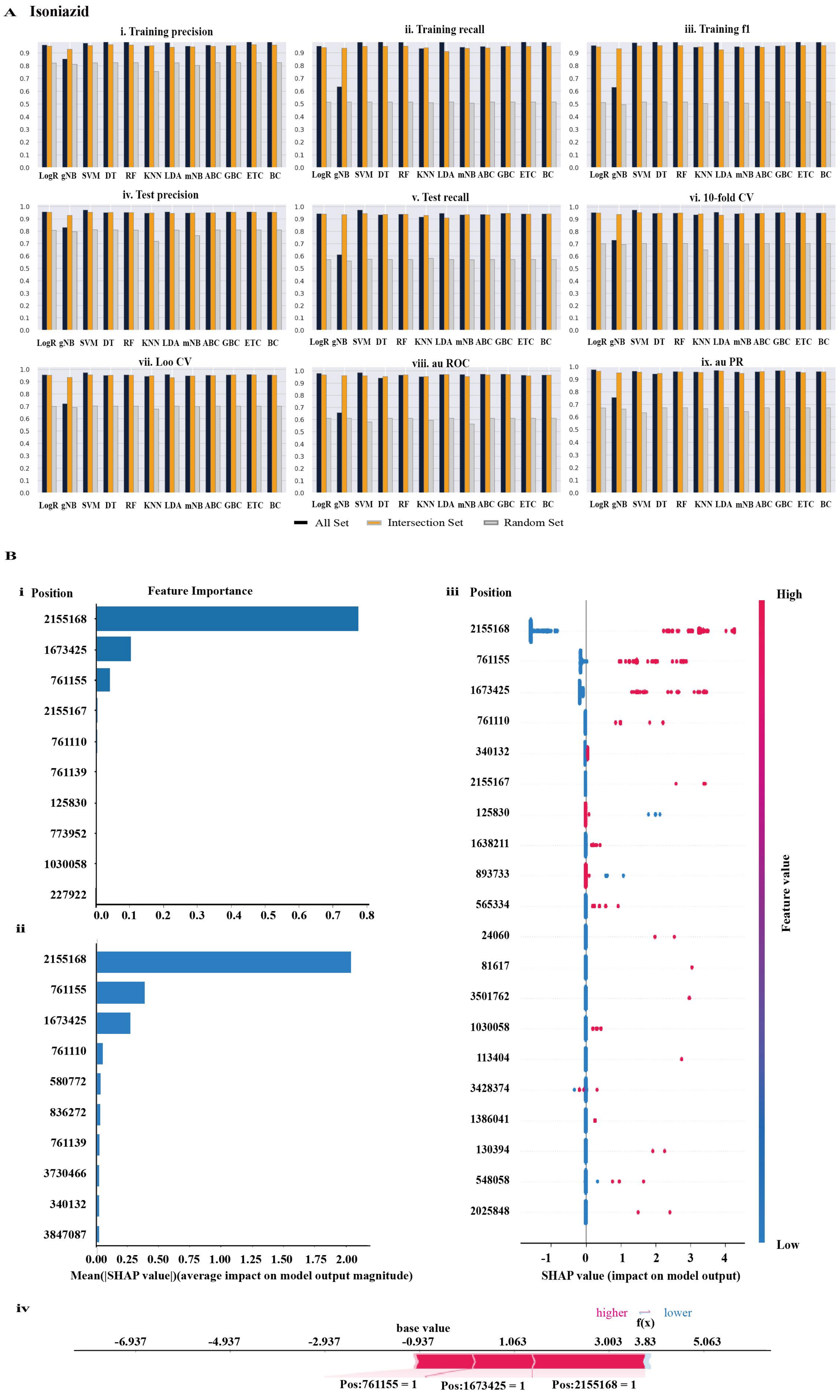

A Isoniazid
i. Training precision
ii. Training recall
iii. Training f1
iv. Test precision
v. Test recall
vi. 10-fold CV
vii. Loo CV
viii. au ROC
ix. au PR
LogR gNB SVM DT RF KNN LDA mNB ABC GBC ETC BC
All Set
Intersection Set
Random Set
B
i Position
Feature Importance
2155168
1673425
761155
2155167
761110
761139
125830
773952
1030058
227922
0.0 0.1 0.2 0.3 0.4 0.5 0.6 0.7 0.8
ii
2155168
761155
1673425
761110
580772
836272
761139
3730466
340132
3847087
0.00 0.25 0.50 0.75 1.00 1.25 1.50 1.75 2.00
Mean(|SHAP value|)(average impact on model output magnitude)
iii Position
2155168
761155
1673425
761110
340132
2155167
125830
1638211
893733
565334
24060
81617
3501762
1030058
113404
3428374
1386041
130394
548058
2025848
-1 0 1 2 3 4
SHAP value (impact on model output)
High
Feature value
Low
iv
higher
lower
f(x)
base value
-6.937 -4.937 -2.937 -0.937 1.063 3.003 3.83 5.063
Pos:761155 = 1
Pos:1673425 = 1
Pos:2155168 = 1

Figure S2

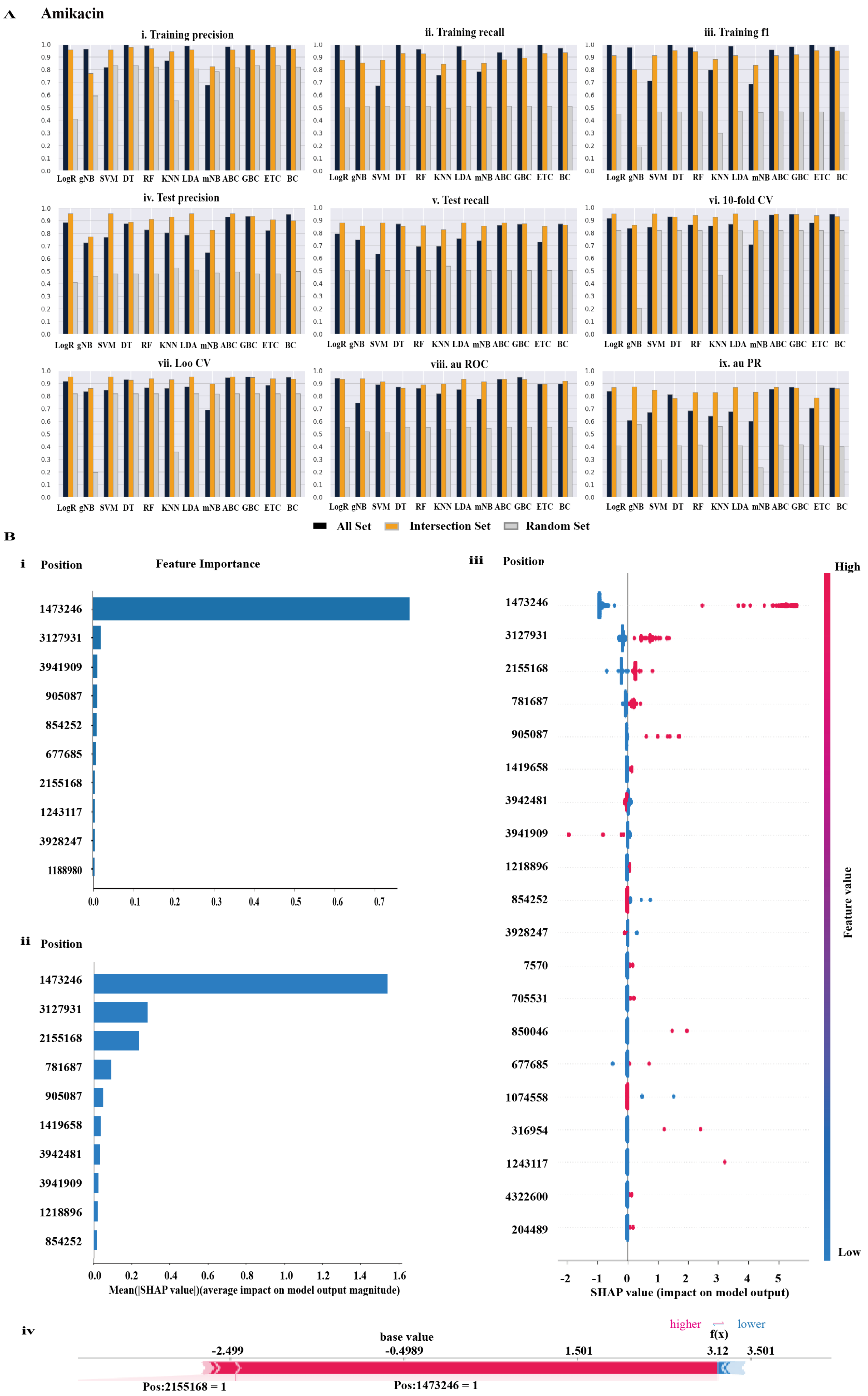

A Amikacin
i. Training precision
ii. Training recall
iii. Training f1
iv. Test precision
v. Test recall
vi. 10-fold CV
vii. Loo CV
viii. au ROC
ix. au PR
LogR gNB SVM DT RF KNN LDA mNB ABC GBC ETC BC
All Set
Intersection Set
Random Set
B
i Position
Feature Importance
1473246
3127931
3941909
905087
854252
677685
2155168
1243117
3928247
1188980
ii Position
1473246
3127931
2155168
781687
905087
1419658
3942481
3941909
1218896
854252
Mean(|SHAP value|)(average impact on model output magnitude)
iii Position
1473246
3127931
2155168
781687
905087
1419658
3942481
3941909
1218896
854252
3928247
7570
705531
850046
677685
1074558
316954
1243117
4322600
204489
SHAP value (impact on model output)
High
Feature value
Low
iv
higher
lower
f(x)
base value
-2.499
-0.4989
1.501
3.12
3.501
Pos:2155168 = 1
Pos:1473246 = 1

Figure S3

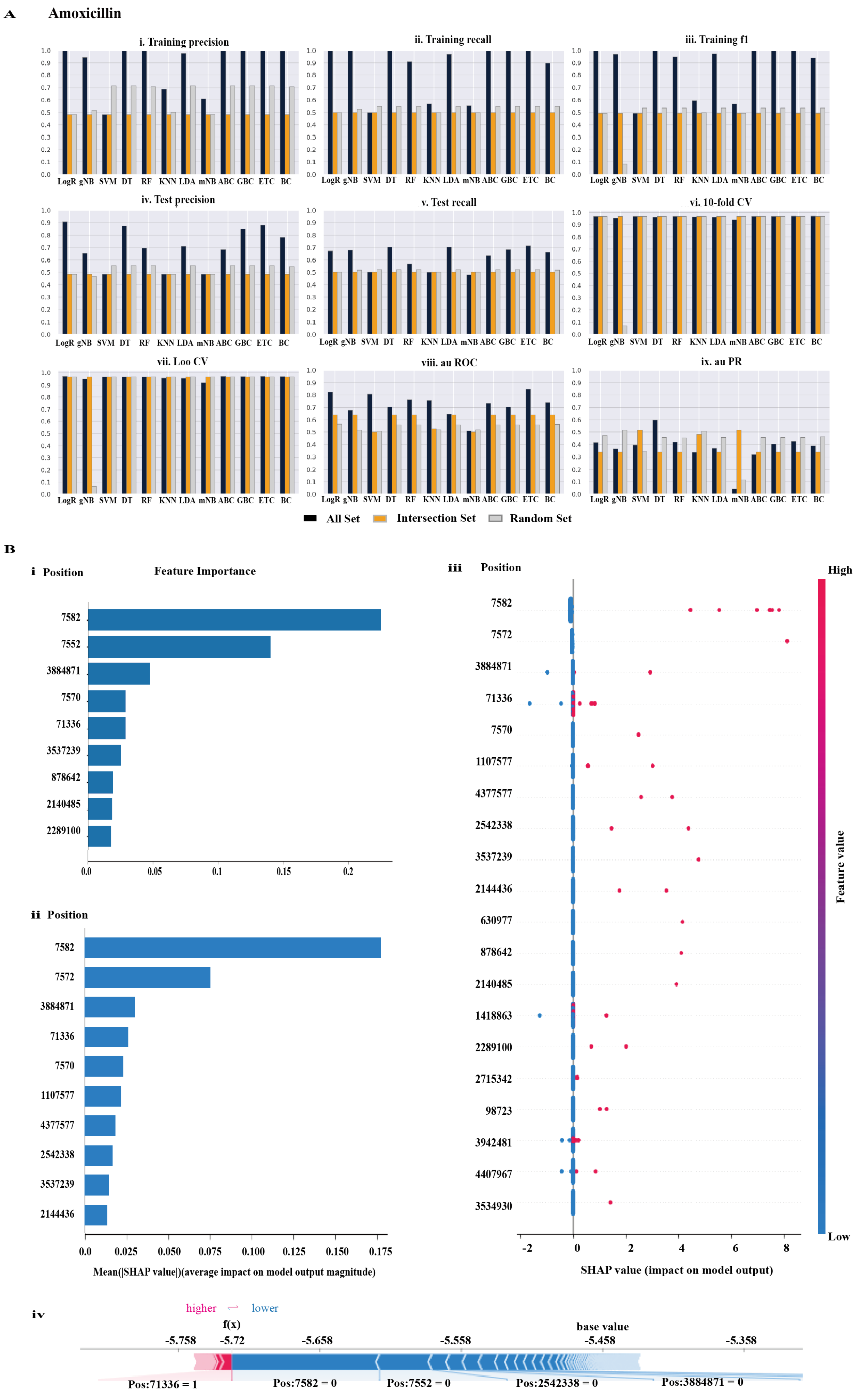
A
Amoxicillin
i. Training precision
ii. Training recall
iii. Training f1
iv. Test precision
v. Test recall
vi. 10-fold CV
vii. Loo CV
viii. au ROC
ix. au PR
LogR gNB SVM DT RF KNN LDA mNB ABC GBC ETC BC
All Set
Intersection Set
Random Set
B
i Position
Feature Importance
7582
7552
3884871
7570
71336
3537239
878642
2140485
2289100
ii Position
7582
7572
3884871
71336
7570
1107577
4377577
2542338
3537239
2144436
Mean(|SHAP value|)(average impact on model output magnitude)
iii Position
7582
7572
3884871
71336
7570
1107577
4377577
2542338
3537239
2144436
630977
878642
2140485
1418863
2289100
2715342
98723
3942481
4407967
3534930
High
Feature value
Low
SHAP value (impact on model output)
iv
higher
lower
f(x)
base value
-5.758
-5.72
-5.658
-5.558
-5.458
-5.358
Pos:71336 = 1
Pos:7582 = 0
Pos:7552 = 0
Pos:2542338 = 0
Pos:3884871 = 0

Figure S4

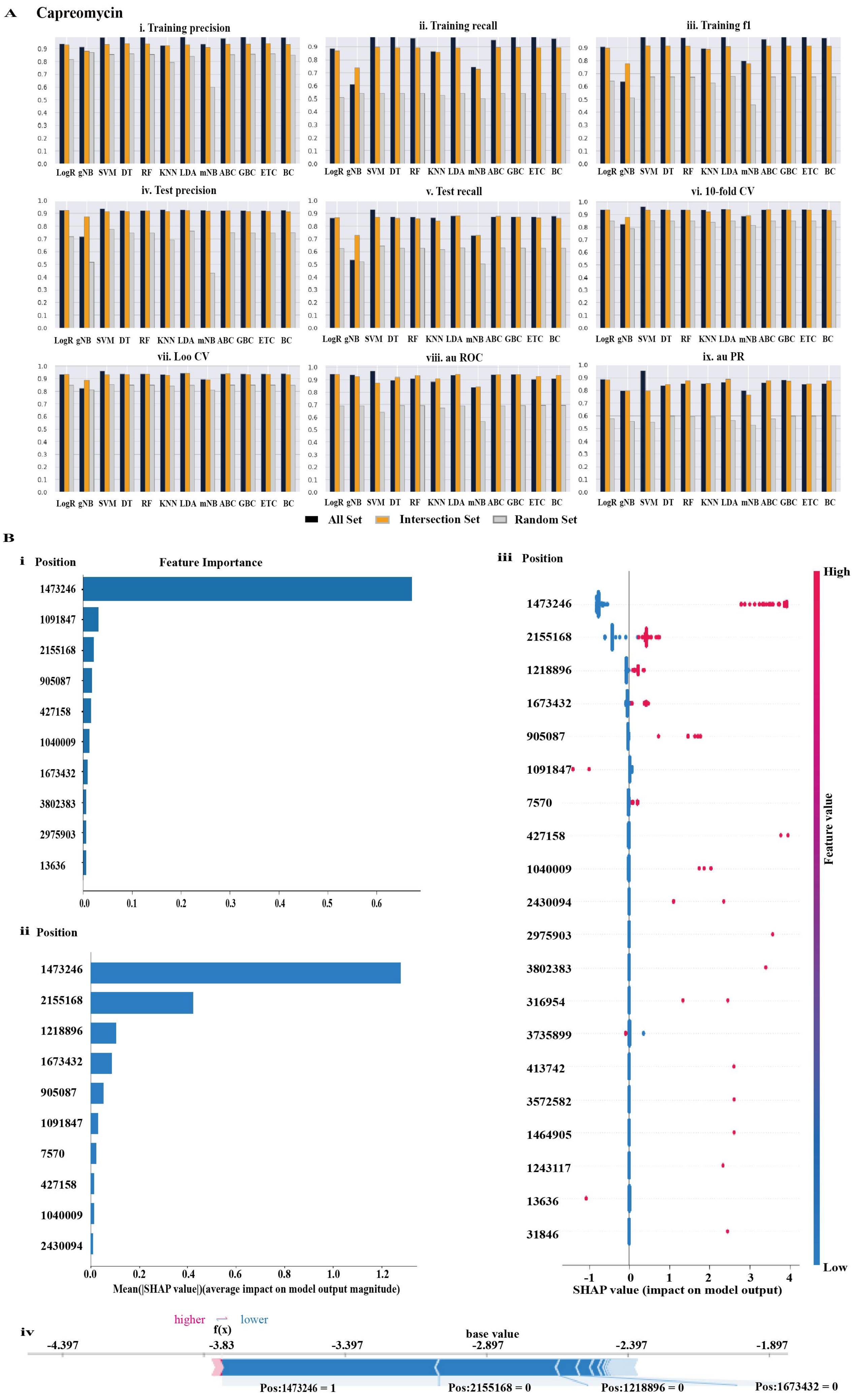

A Capreomycin
i. Training precision
ii. Training recall
iii. Training f1
iv. Test precision
v. Test recall
vi. 10-fold CV
vii. Loo CV
viii. au ROC
ix. au PR
LogR gNB SVM DT RF KNN LDA mNB ABC GBC ETC BC
All Set
Intersection Set
Random Set
B
i Position
Feature Importance
1473246
1091847
2155168
905087
427158
1040009
1673432
3802383
2975903
13636
ii Position
1473246
2155168
1218896
1673432
905087
1091847
7570
427158
1040009
2430094
Mean(|SHAP value|)(average impact on model output magnitude)
iii Position
1473246
2155168
1218896
1673432
905087
1091847
7570
427158
1040009
2430094
2975903
3802383
316954
3735899
413742
3572582
1464905
1243117
13636
31846
SHAP value (impact on model output)
High
Feature value
Low
iv
higher
lower
f(x)
base value
-4.397
-3.83
-3.397
-2.897
-2.397
-1.897
Pos:1473246 = 1
Pos:2155168 = 0
Pos:1218896 = 0
Pos:1673432 = 0

Figure S5

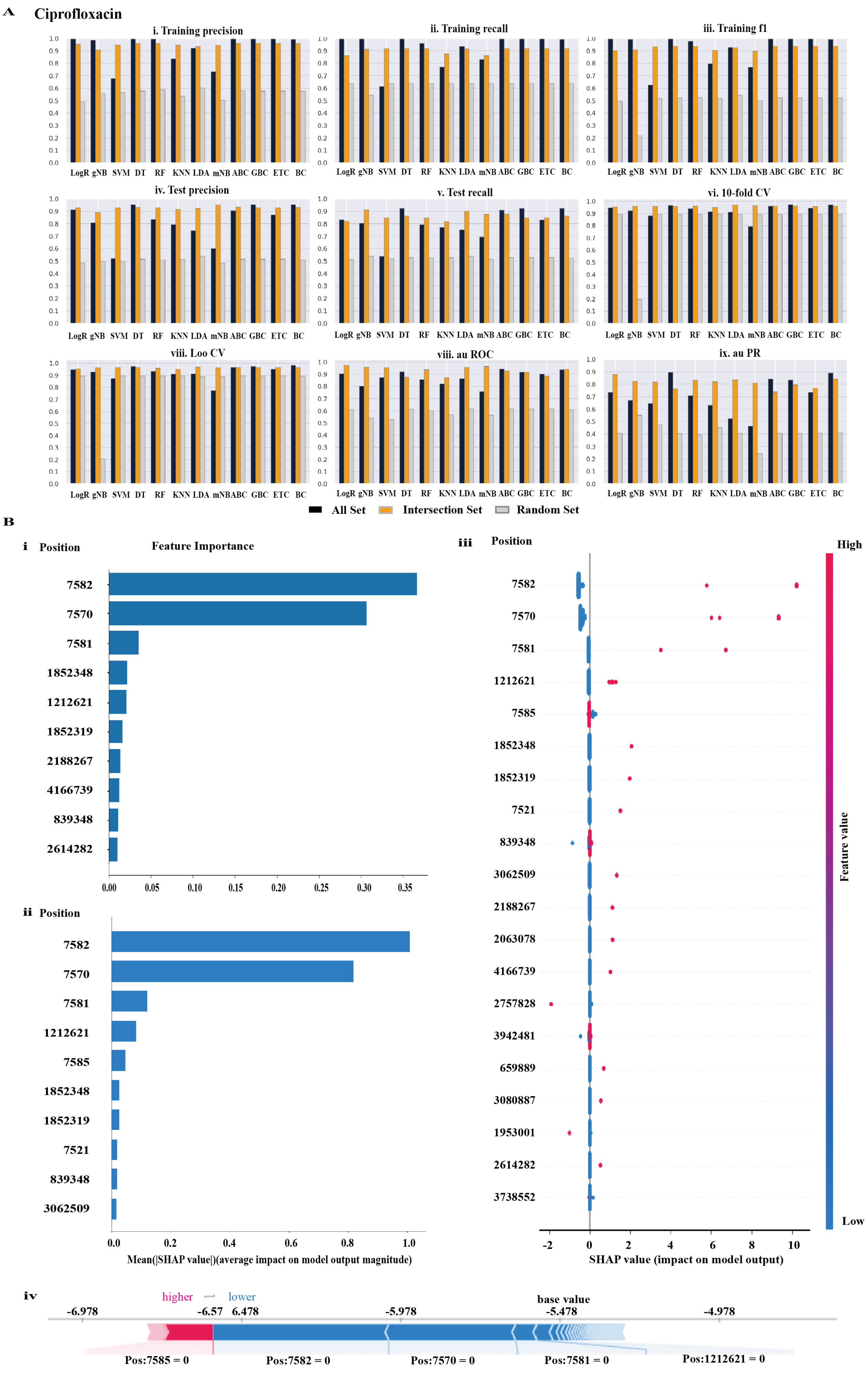
A Ciprofloxacin
i. Training precision
ii. Training recall
iii. Training f1
iv. Test precision
v. Test recall
vi. 10-fold CV
viii. Loo CV
viii. au ROC
ix. au PR
LogR gNB SVM DT RF KNN LDA mNB ABC GBC ETC BC
All Set
Intersection Set
Random Set
B
i Position
Feature Importance
7582
7570
7581
1852348
1212621
1852319
2188267
4166739
839348
2614282
ii Position
7585
7521
3062509
Mean(|SHAP value|)(average impact on model output magnitude)
iii Position
2063078
2757828
3942481
659889
3080887
1953001
3738552
SHAP value (impact on model output)
High
Low
Feature value
iv
higher
lower
base value
-6.978
-6.57
6.478
-5.978
-5.478
-4.978
Pos:7585 = 0
Pos:7582 = 0
Pos:7570 = 0
Pos:7581 = 0
Pos:1212621 = 0

Figure S6

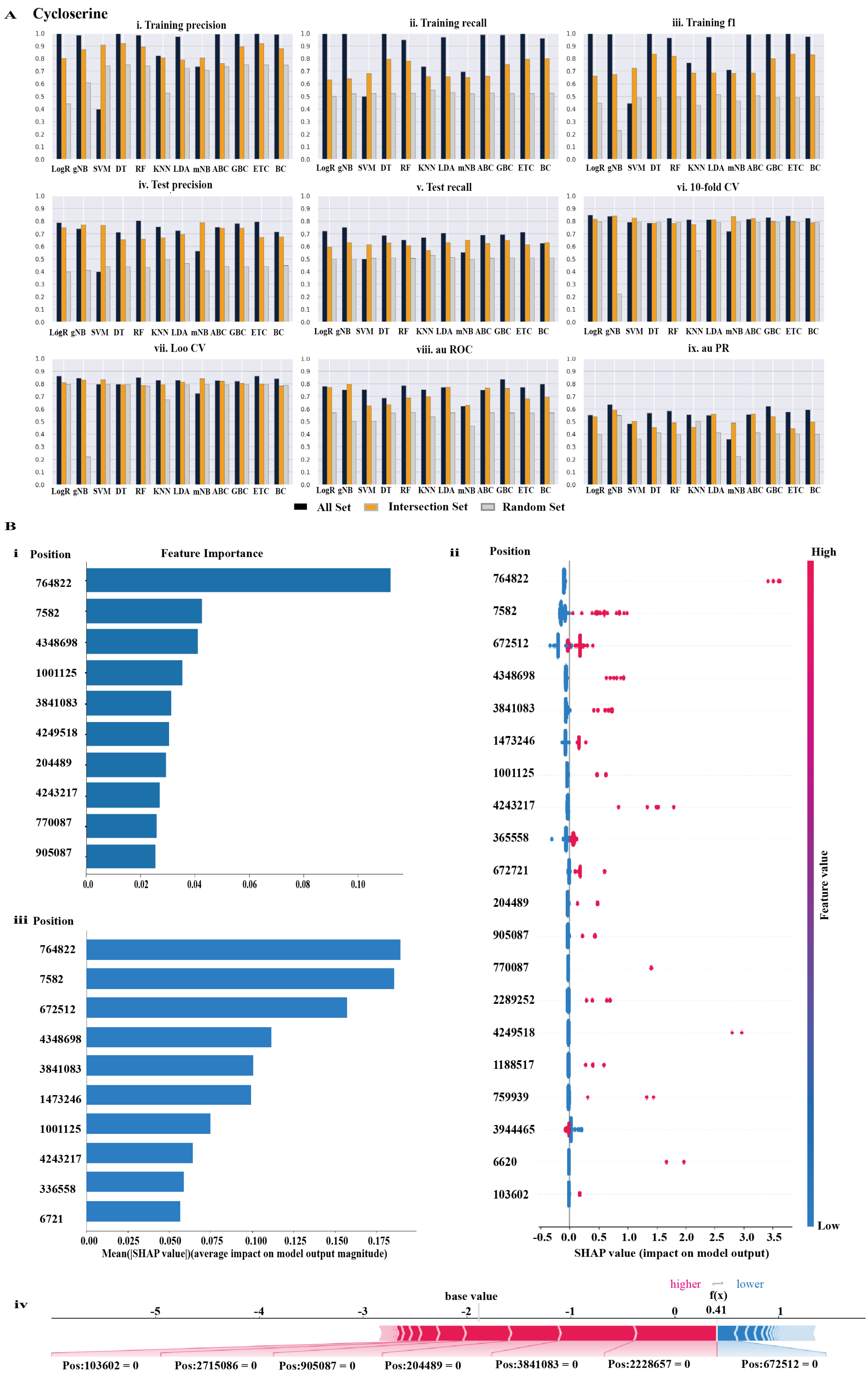
A
Cycloserine
i. Training precision
ii. Training recall
iii. Training f1
iv. Test precision
v. Test recall
vi. 10-fold CV
vii. Loo CV
viii. au ROC
ix. au PR
LogR gNB SVM DT RF KNN LDA mNB ABC GBC ETC BC
All Set
Intersection Set
Random Set
B
i
Position
Feature Importance
764822
7582
4348698
1001125
3841083
4249518
204489
4243217
770087
905087
0.0 0.02 0.04 0.06 0.08 0.10
ii
Position
764822
7582
672512
4348698
3841083
1473246
1001125
4243217
365558
672721
204489
905087
770087
2289252
4249518
1188517
759939
3944465
6620
103602
High
Feature value
Low
-0.5 0.0 0.5 1.0 1.5 2.0 2.5 3.0 3.5
SHAP value (impact on model output)
iii
Position
764822
7582
672512
4348698
3841083
1473246
1001125
4243217
336558
6721
0.00 0.025 0.050 0.075 0.100 0.125 0.150 0.175
Mean(|SHAP value|)(average impact on model output magnitude)
iv
higher
lower
base value
f(x)
0.41
-5 -4 -3 -2 -1 0 1
Pos:103602 = 0
Pos:2715086 = 0
Pos:905087 = 0
Pos:204489 = 0
Pos:3841083 = 0
Pos:2228657 = 0
Pos:672512 = 0

Figure S7

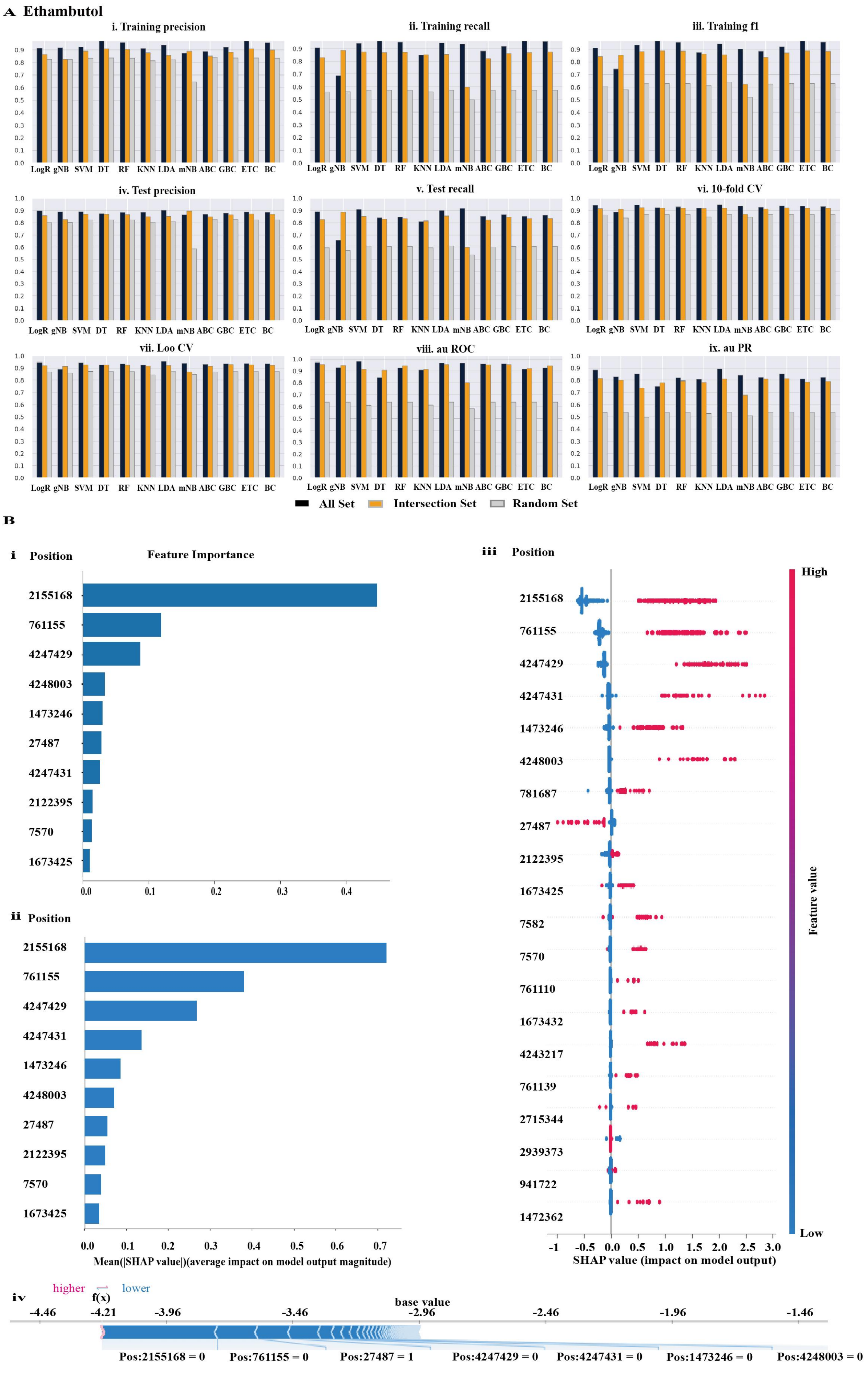
A Ethambutol
i. Training precision
ii. Training recall
iii. Training f1
iv. Test precision
v. Test recall
vi. 10-fold CV
vii. Loo CV
viii. au ROC
ix. au PR
LogR gNB SVM DT RF KNN LDA mNB ABC GBC ETC BC
All Set
Intersection Set
Random Set
B
i Position
Feature Importance
2155168
761155
4247429
4248003
1473246
27487
4247431
2122395
7570
1673425
ii Position
2155168
761155
4247429
4247431
1473246
4248003
27487
2122395
7570
1673425
Mean(|SHAP value|)(average impact on model output magnitude)
iii Position
2155168
761155
4247429
4247431
1473246
4248003
781687
27487
2122395
1673425
7582
7570
761110
1673432
4243217
761139
2715344
2939373
941722
1472362
High
Feature value
Low
SHAP value (impact on model output)
iv
higher
lower
f(x)
base value
-4.46
-4.21
-3.96
-3.46
-2.96
-2.46
-1.96
-1.46
Pos:2155168 = 0
Pos:761155 = 0
Pos:27487 = 1
Pos:4247429 = 0
Pos:4247431 = 0
Pos:1473246 = 0
Pos:4248003 = 0

Figure S8

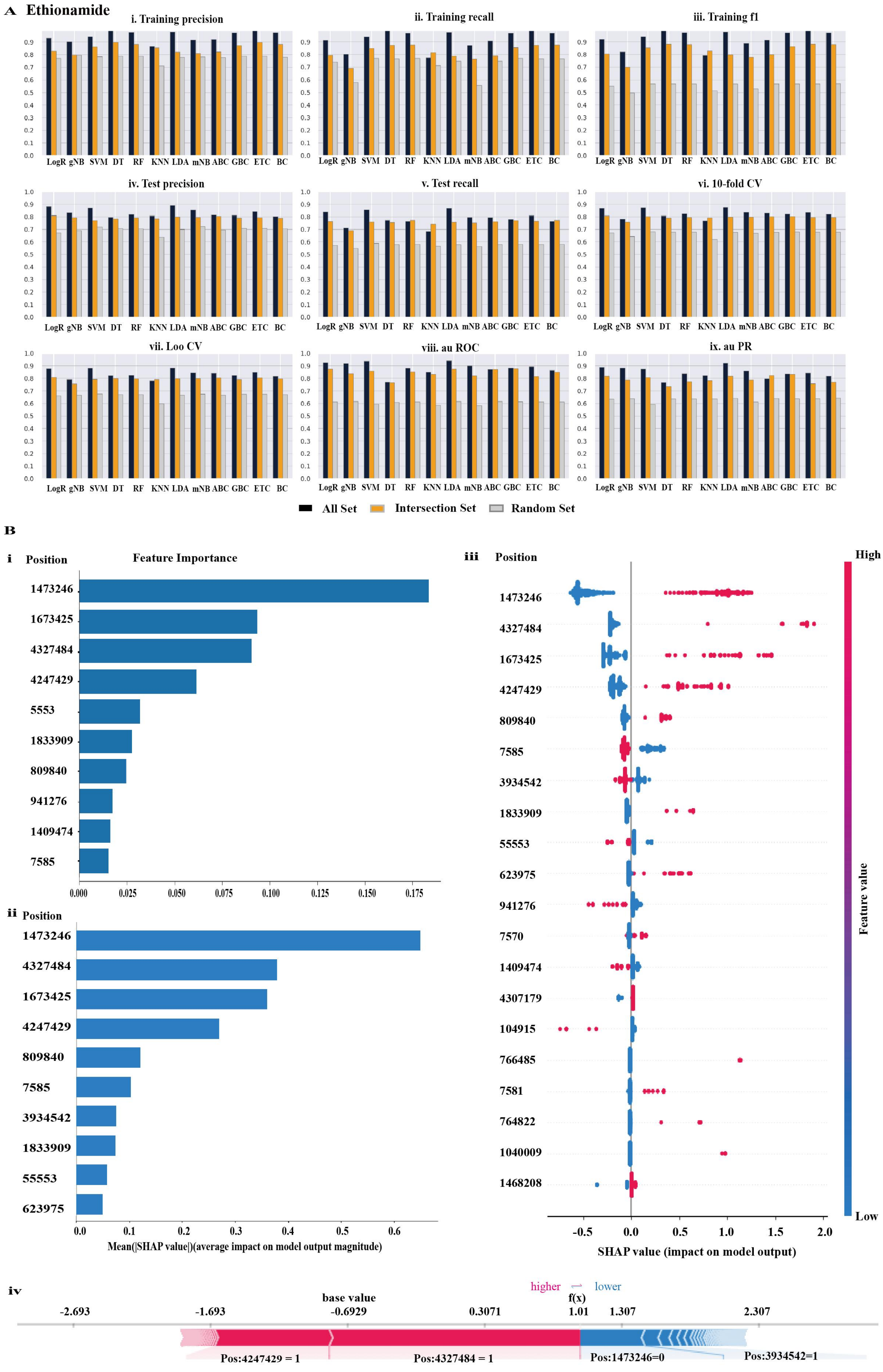
A Ethionamide
i. Training precision
ii. Training recall
iii. Training f1
iv. Test precision
v. Test recall
vi. 10-fold CV
vii. Loo CV
viii. au ROC
ix. au PR
LogR gNB SVM DT RF KNN LDA mNB ABC GBC ETC BC
All Set
Intersection Set
Random Set
B
i Position
Feature Importance
1473246
1673425
4327484
4247429
5553
1833909
809840
941276
1409474
7585
0.000 0.025 0.050 0.075 0.100 0.125 0.150 0.175
ii Position
1473246
4327484
1673425
4247429
809840
7585
3934542
1833909
55553
623975
0.0 0.1 0.2 0.3 0.4 0.5 0.6
Mean(|SHAP value|)(average impact on model output magnitude)
iii Position
1473246
4327484
1673425
4247429
809840
7585
3934542
1833909
55553
623975
941276
7570
1409474
4307179
104915
766485
7581
764822
1040009
1468208
-0.5 0.0 0.5 1.0 1.5 2.0
SHAP value (impact on model output)
High
Feature value
Low
iv
higher
lower
base value
f(x)
-2.693
-1.693
-0.6929
0.3071
1.01
1.307
2.307
Pos:4247429 = 1
Pos:4327484 = 1
Pos:1473246=0
Pos:3934542=1

Figure S9

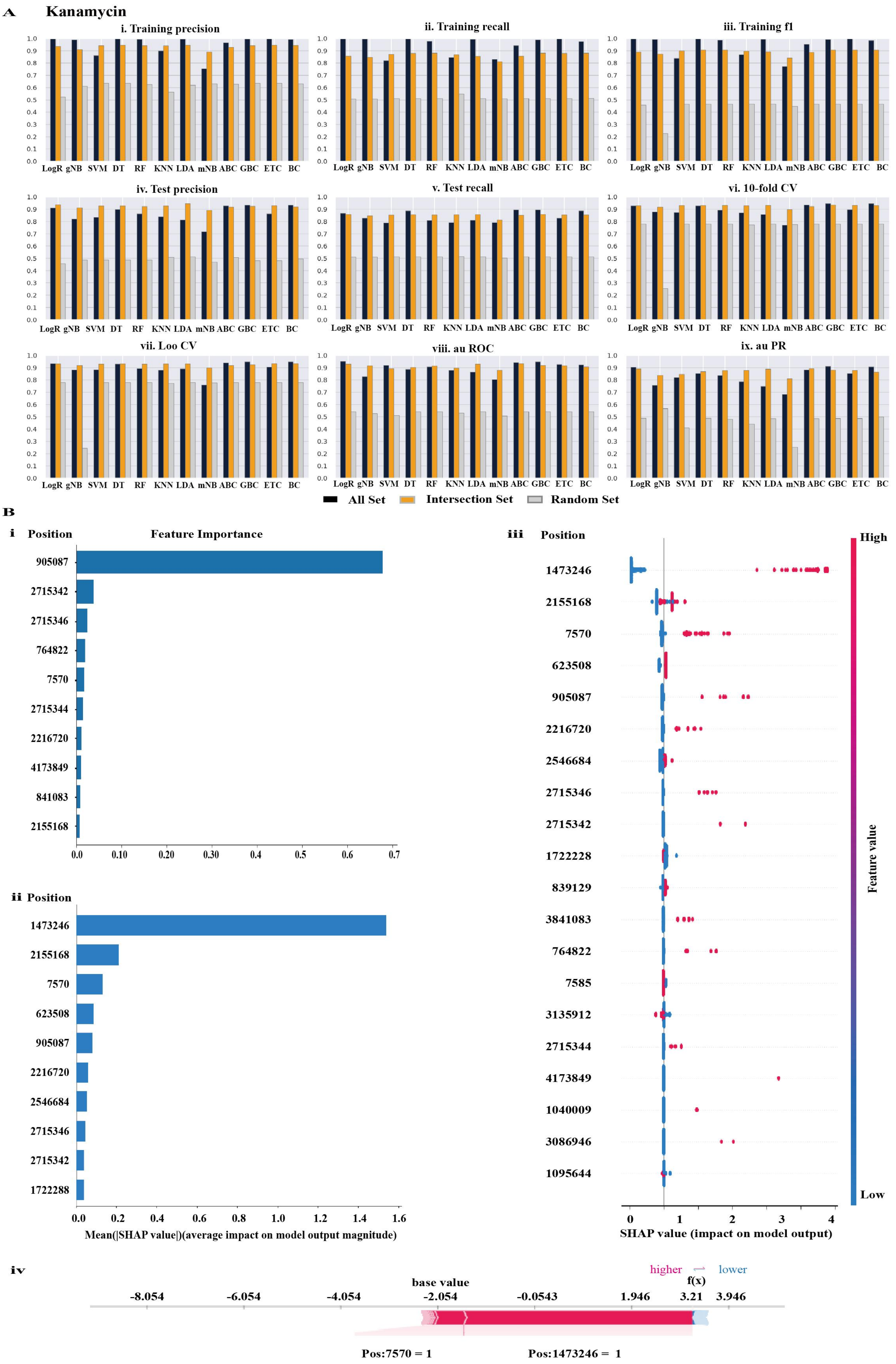
A
Kanamycin
i. Training precision
ii. Training recall
iii. Training f1
iv. Test precision
v. Test recall
vi. 10-fold CV
vii. Loo CV
viii. au ROC
ix. au PR
LogR gNB SVM DT RF KNN LDA mNB ABC GBC ETC BC
All Set
Intersection Set
Random Set
B
i Position
Feature Importance
905087
2715342
2715346
764822
7570
2715344
2216720
4173849
841083
2155168
ii Position
1473246
2155168
7570
623508
905087
2216720
2546684
2715346
2715342
1722288
Mean(|SHAP value|)(average impact on model output magnitude)
iii Position
1473246
2155168
7570
623508
905087
2216720
2546684
2715346
2715342
1722228
839129
3841083
764822
7585
3135912
2715344
4173849
1040009
3086946
1095644
High
Feature value
Low
SHAP value (impact on model output)
iv
higher
lower
base value
f(x)
-8.054
-6.054
-4.054
-2.054
-0.0543
1.946
3.21
3.946
Pos:7570 = 1
Pos:1473246 = 1

Figure S10

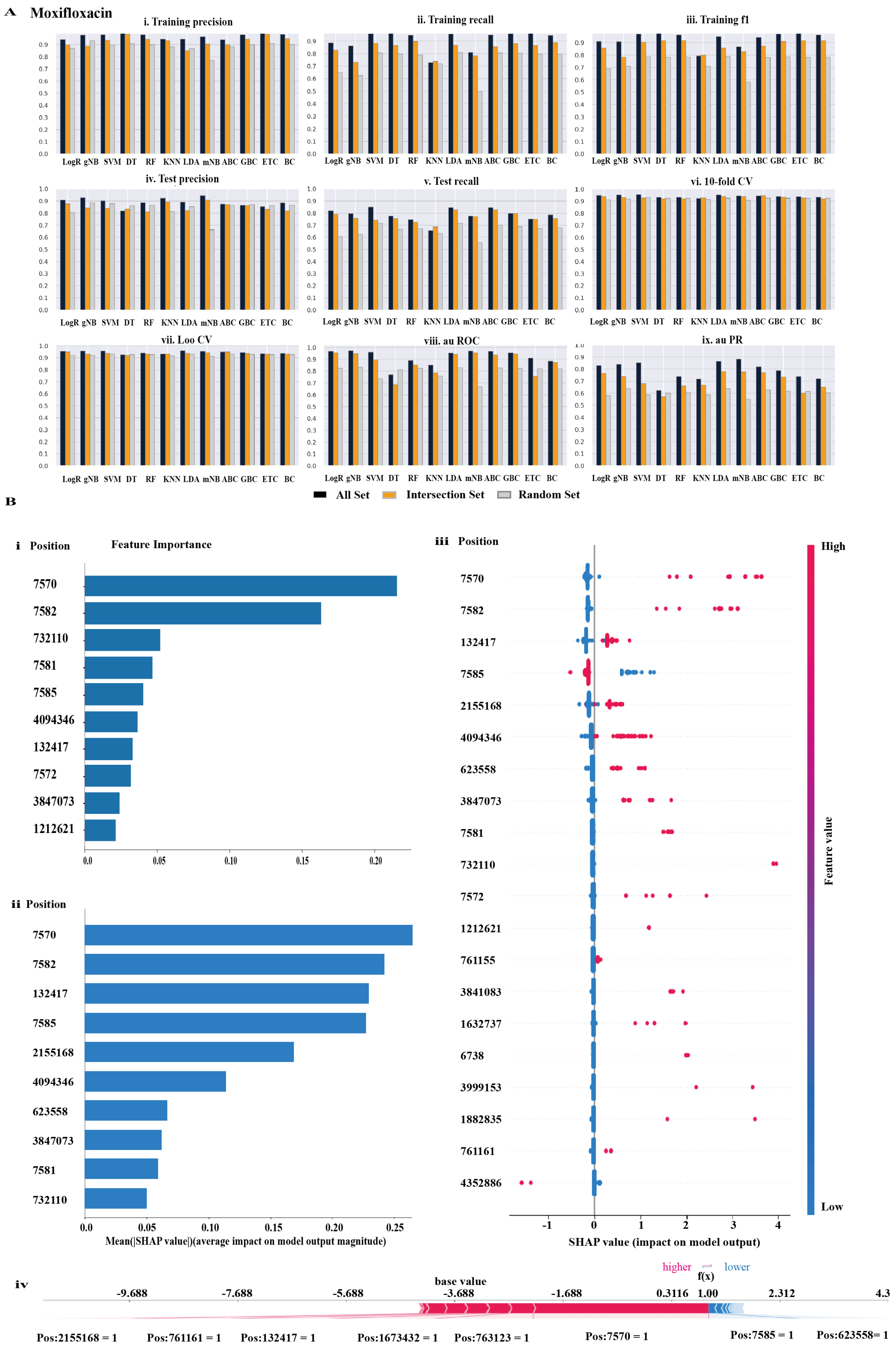

A Moxifloxacin
i. Training precision
ii. Training recall
iii. Training f1
iv. Test precision
v. Test recall
vi. 10-fold CV
vii. Loo CV
viii. au ROC
ix. au PR
LogR gNB SVM DT RF KNN LDA mNB ABC GBC ETC BC
All Set
Intersection Set
Random Set
B
i Position
Feature Importance
7570
7582
732110
7581
7585
4094346
132417
7572
3847073
1212621
ii Position
7570
7582
132417
7585
2155168
4094346
623558
3847073
7581
732110
Mean(|SHAP value|)(average impact on model output magnitude)
iii Position
7570
7582
132417
7585
2155168
4094346
623558
3847073
7581
732110
7572
1212621
761155
3841083
1632737
6738
3999153
1882835
761161
4352886
SHAP value (impact on model output)
High
Feature value
Low
iv
higher
lower
f(x)
base value
-9.688
-7.688
-5.688
-3.688
-1.688
0.3116
1.00
2.312
4.3
Pos:2155168 = 1
Pos:761161 = 1
Pos:132417 = 1
Pos:1673432 = 1
Pos:763123 = 1
Pos:7570 = 1
Pos:7585 = 1
Pos:623558= 1

Figure S11

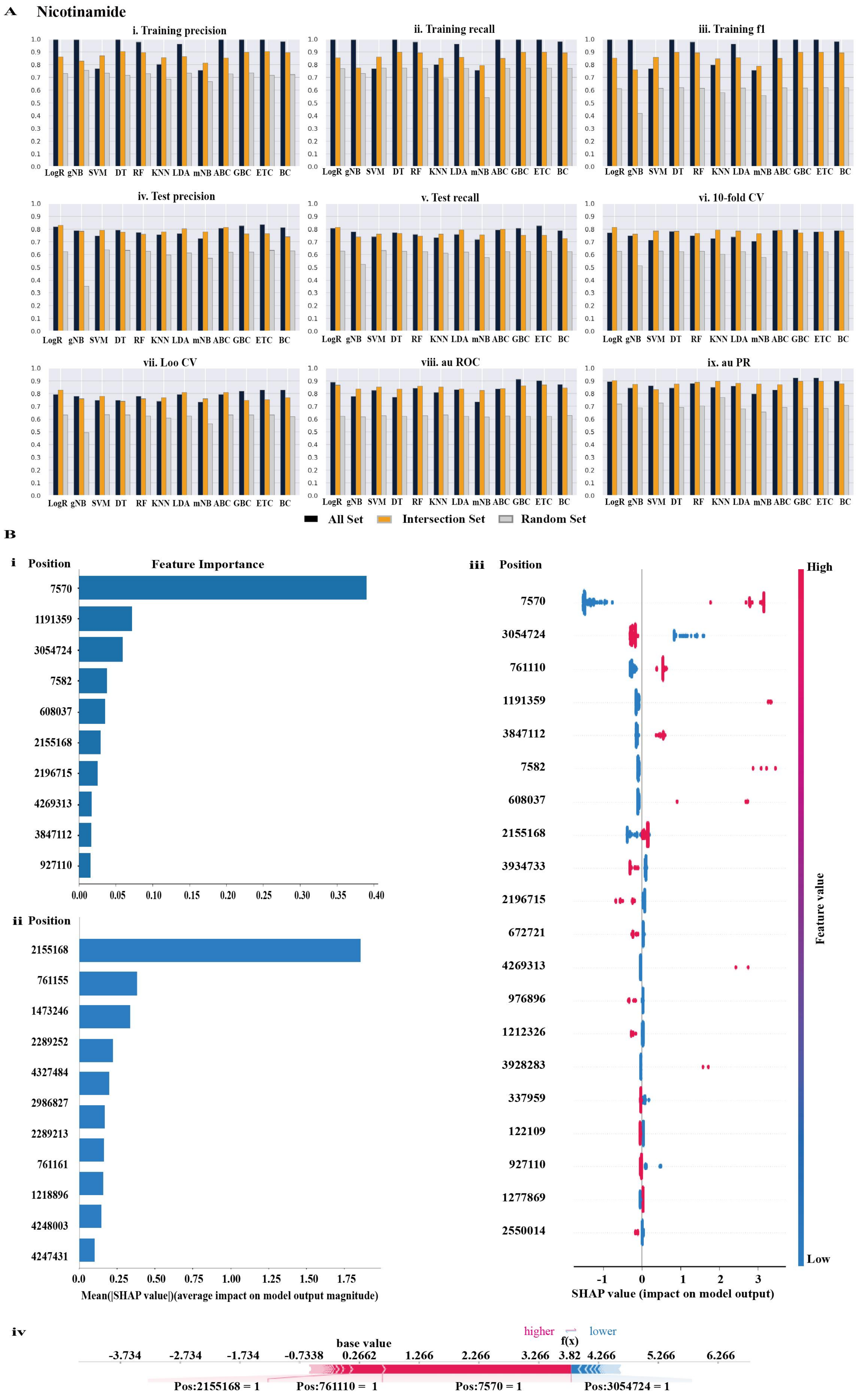
A Nicotinamide
i. Training precision
ii. Training recall
iii. Training f1
iv. Test precision
v. Test recall
vi. 10-fold CV
vii. Loo CV
viii. au ROC
ix. au PR
LogR gNB SVM DT RF KNN LDA mNB ABC GBC ETC BC
All Set
Intersection Set
Random Set
B
i Position
Feature Importance
7570
1191359
3054724
7582
608037
2155168
2196715
4269313
3847112
927110
0.00 0.05 0.10 0.15 0.20 0.25 0.30 0.35 0.40
ii Position
2155168
761155
1473246
2289252
4327484
2986827
2289213
761161
1218896
4248003
4247431
0.0 0.25 0.50 0.75 1.00 1.25 1.50 1.75
Mean(|SHAP value|)(average impact on model output magnitude)
iii Position
7570
3054724
761110
1191359
3847112
7582
608037
2155168
3934733
2196715
672721
4269313
976896
1212326
3928283
337959
122109
927110
1277869
2550014
-1 0 1 2 3
SHAP value (impact on model output)
High
Feature value
Low
iv
higher
lower
base value
f(x)
-3.734 -2.734 -1.734 -0.7338 0.2662 1.266 2.266 3.266 3.82 4.266 5.266 6.266
Pos:2155168 = 1
Pos:761110 = 1
Pos:7570 = 1
Pos:3054724 = 1

Figure S12

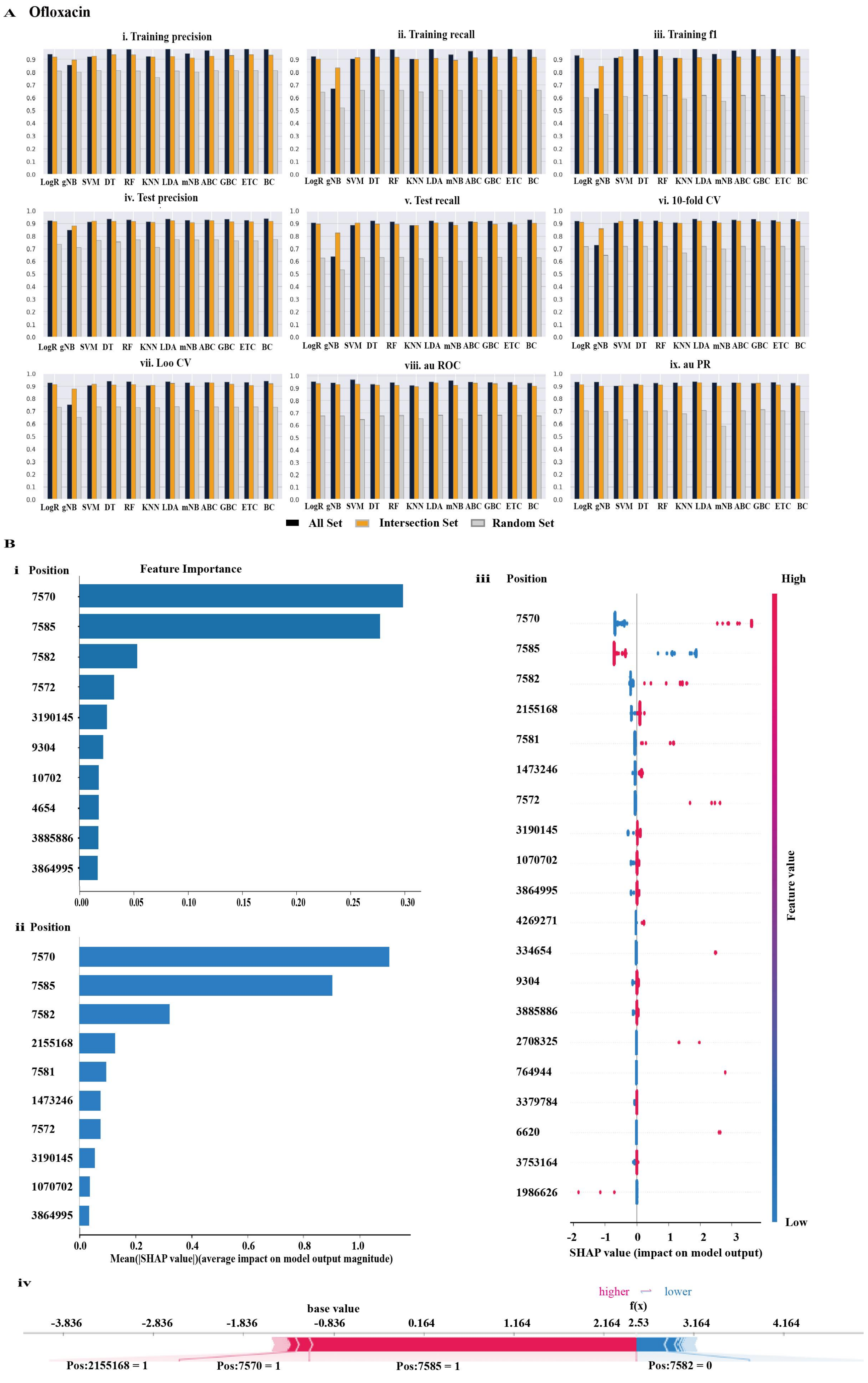
A Ofloxacin
i. Training precision
ii. Training recall
iii. Training f1
iv. Test precision
v. Test recall
vi. 10-fold CV
vii. Loo CV
viii. au ROC
ix. au PR
LogR gNB SVM DT RF KNN LDA mNB ABC GBC ETC BC
All Set
Intersection Set
Random Set
B
i Position
Feature Importance
7570
7585
7582
7572
3190145
9304
10702
4654
3885886
3864995
0.00 0.05 0.10 0.15 0.20 0.25 0.30
ii Position
7570
7585
7582
2155168
7581
1473246
7572
3190145
1070702
3864995
0.0 0.2 0.4 0.6 0.8 1.0
Mean(|SHAP value|)(average impact on model output magnitude)
iii Position
High
7570
7585
7582
2155168
7581
1473246
7572
3190145
1070702
3864995
4269271
334654
9304
3885886
2708325
764944
3379784
6620
3753164
1986626
Feature value
Low
-2 -1 0 1 2 3
SHAP value (impact on model output)
iv
higher
lower
f(x)
base value
-3.836
-2.836
-1.836
-0.836
0.164
1.164
2.164
2.53
3.164
4.164
Pos:2155168 = 1
Pos:7570 = 1
Pos:7585 = 1
Pos:7582 = 0

Figure S13

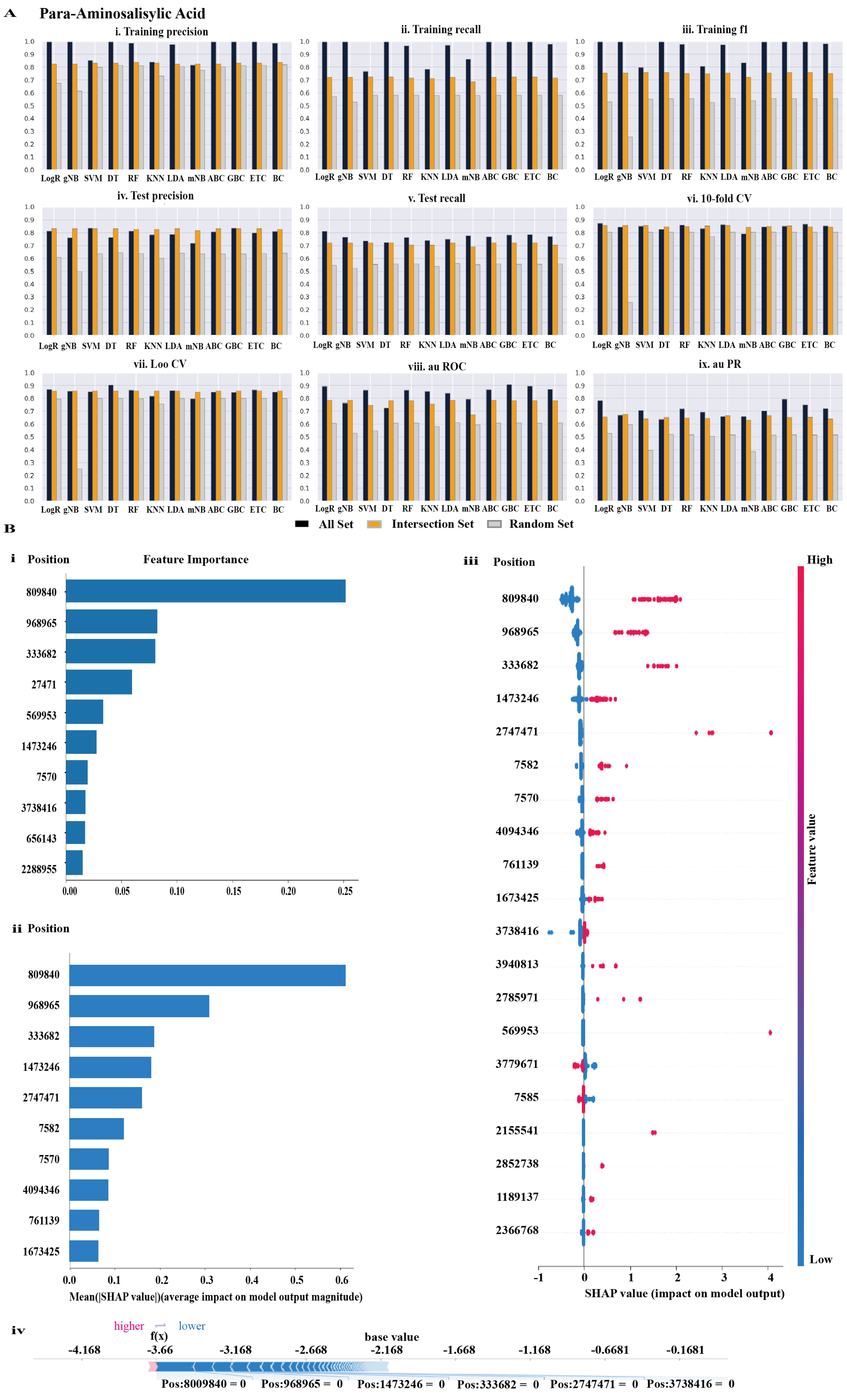
A
Para-Aminosalisylic Acid
i. Training precision
ii. Training recall
iii. Training f1
iv. Test precision
v. Test recall
vi. 10-fold CV
vii. Loo CV
viii. au ROC
ix. au PR
LogR gNB SVM DT RF KNN LDA mNB ABC GBC ETC BC
All Set
Intersection Set
Random Set
B
i Position
Feature Importance
809840
968965
333682
27471
569953
1473246
7570
3738416
656143
2288955
ii Position
809840
968965
333682
1473246
2747471
7582
7570
4094346
761139
1673425
Mean(|SHAP value|)(average impact on model output magnitude)
iii Position
809840
968965
333682
1473246
2747471
7582
7570
4094346
761139
1673425
3738416
3940813
2785971
569953
3779671
7585
2155541
2852738
1189137
2366768
High
Feature value
Low
SHAP value (impact on model output)
iv
higher
lower
f(x)
base value
-4.168
-3.66
-3.168
-2.668
-2.168
-1.668
-1.168
-0.6681
-0.1681
Pos:8009840 = 0
Pos:968965 = 0
Pos:1473246 = 0
Pos:333682 = 0
Pos:2747471 = 0
Pos:3738416 = 0

Figure S14

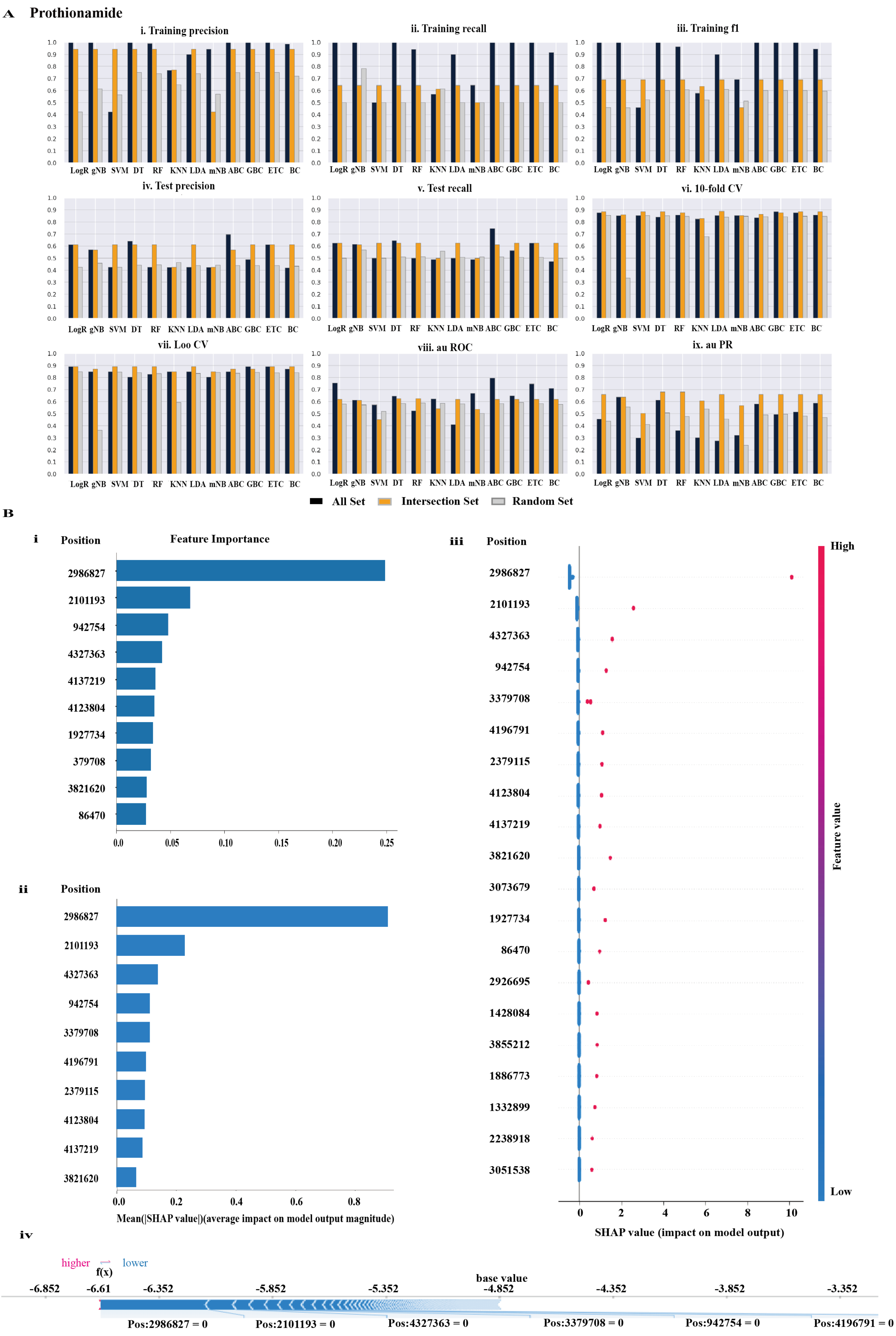
A Prothionamide
i. Training precision
ii. Training recall
iii. Training f1
iv. Test precision
v. Test recall
vi. 10-fold CV
vii. Loo CV
viii. au ROC
ix. au PR
LogR gNB SVM DT RF KNN LDA mNB ABC GBC ETC BC
All Set
Intersection Set
Random Set
B
i Position Feature Importance
ii Position
Mean(|SHAP value|)(average impact on model output magnitude)
iii Position
SHAP value (impact on model output)
High
Feature value
Low
iv
higher
lower
f(x)
base value
Pos:2986827 = 0
Pos:2101193 = 0
Pos:4327363 = 0
Pos:3379708 = 0
Pos:942754 = 0
Pos:4196791 = 0

Figure S15

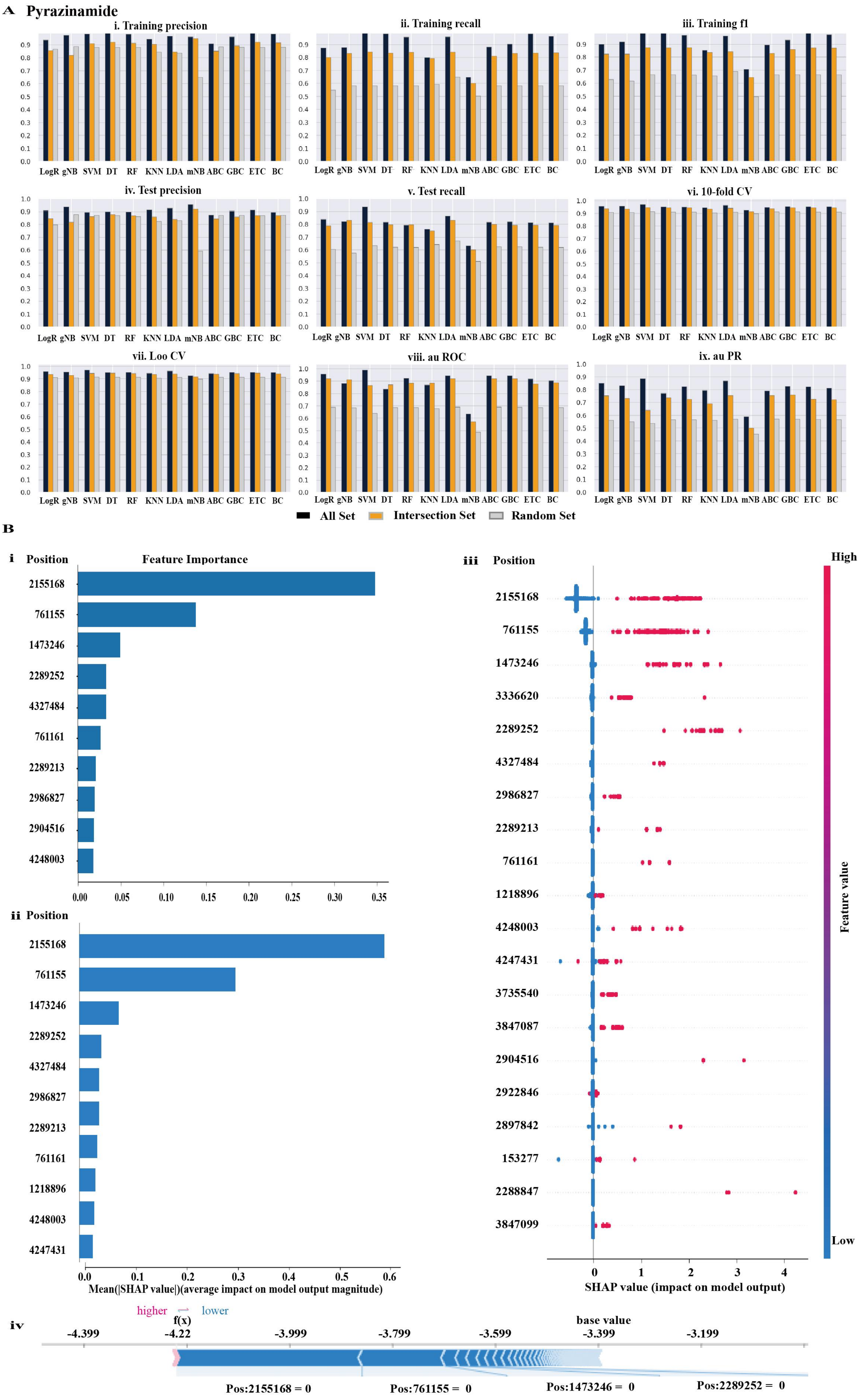
A Pyrazinamide
i. Training precision
ii. Training recall
iii. Training f1
iv. Test precision
v. Test recall
vi. 10-fold CV
vii. Loo CV
viii. au ROC
ix. au PR
LogR gNB SVM DT RF KNN LDA mNB ABC GBC ETC BC
All Set
Intersection Set
Random Set
B
i Position
Feature Importance
2155168
761155
1473246
2289252
4327484
761161
2289213
2986827
2904516
4248003
0.00 0.05 0.10 0.15 0.20 0.25 0.30 0.35
ii Position
2155168
761155
1473246
2289252
4327484
2986827
2289213
761161
1218896
4248003
4247431
0.0 0.1 0.2 0.3 0.4 0.5 0.6
Mean(|SHAP value|)(average impact on model output magnitude)
iii Position
2155168
761155
1473246
3336620
2289252
4327484
2986827
2289213
761161
1218896
4248003
4247431
3735540
3847087
2904516
2922846
2897842
153277
2288847
3847099
0 1 2 3 4
SHAP value (impact on model output)
High
Feature value
Low
iv
higher
lower
f(x)
base value
-4.399 -4.22 -3.999 -3.799 -3.599 -3.399 -3.199
Pos:2155168 = 0
Pos:761155 = 0
Pos:1473246 = 0
Pos:2289252 = 0

Figure S16

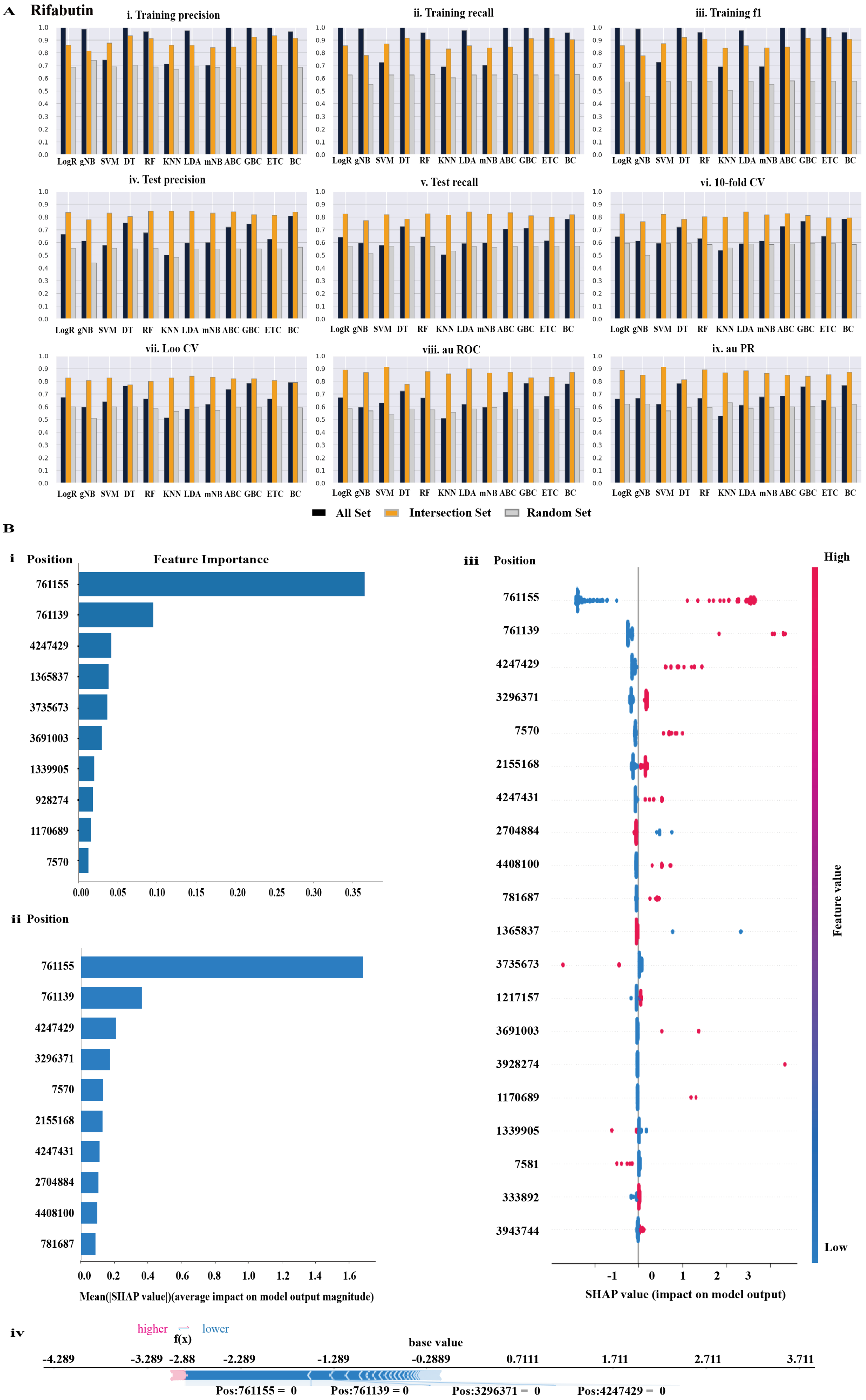
A Rifabutin
i. Training precision
ii. Training recall
iii. Training f1
iv. Test precision
v. Test recall
vi. 10-fold CV
vii. Loo CV
viii. au ROC
ix. au PR
LogR gNB SVM DT RF KNN LDA mNB ABC GBC ETC BC
All Set
Intersection Set
Random Set
B
i Position
Feature Importance
761155
761139
4247429
1365837
3735673
3691003
1339905
928274
1170689
7570
ii Position
761155
761139
4247429
3296371
7570
2155168
4247431
2704884
4408100
781687
Mean(|SHAP value|)(average impact on model output magnitude)
iii Position
761155
761139
4247429
3296371
7570
2155168
4247431
2704884
4408100
781687
1365837
3735673
1217157
3691003
3928274
1170689
1339905
7581
333892
3943744
SHAP value (impact on model output)
High
Feature value
Low
iv
higher
lower
f(x)
base value
-4.289
-3.289
-2.88
-2.289
-1.289
-0.2889
0.7111
1.711
2.711
3.711
Pos:761155 = 0
Pos:761139 = 0
Pos:3296371 = 0
Pos:4247429 = 0

Figure S17

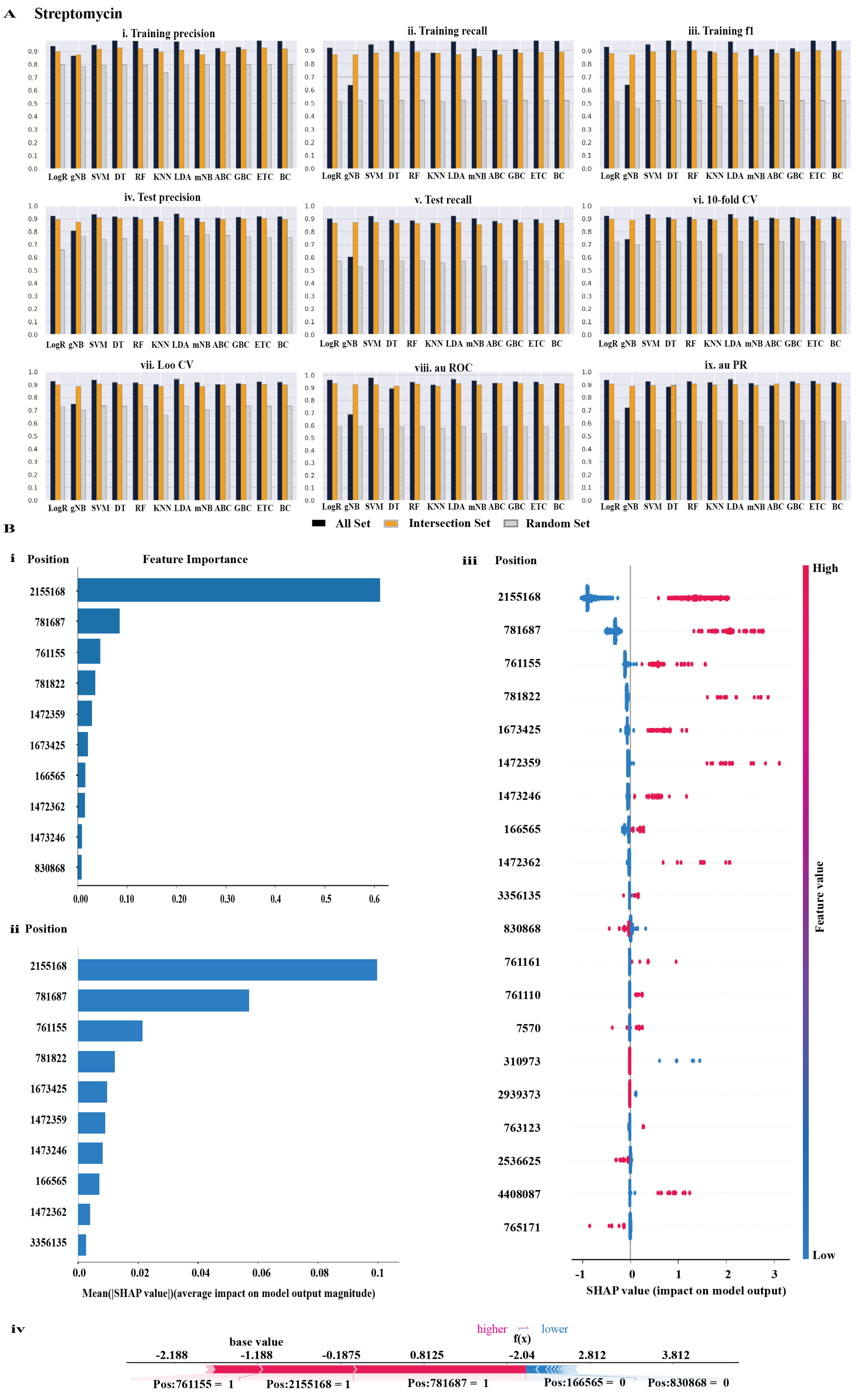
A Streptomycin
i. Training precision
ii. Training recall
iii. Training f1
iv. Test precision
v. Test recall
vi. 10-fold CV
vii. Loo CV
viii. au ROC
ix. au PR
LogR gNB SVM DT RF KNN LDA mNB ABC GBC ETC BC
All Set
Intersection Set
Random Set
B
i Position
Feature Importance
2155168
781687
761155
781822
1472359
1673425
166565
1472362
1473246
830868
0.00 0.10 0.20 0.30 0.4 0.5 0.6
ii Position
2155168
781687
761155
781822
1673425
1472359
1473246
166565
1472362
3356135
0.0 0.02 0.04 0.06 0.08 0.1
Mean(|SHAP value|)(average impact on model output magnitude)
iii Position
2155168
781687
761155
781822
1673425
1472359
1473246
166565
1472362
3356135
830868
761161
761110
7570
310973
2939373
763123
2536625
4408087
765171
-1 0 1 2 3
SHAP value (impact on model output)
High
Feature value
Low
iv
higher
lower
base value
f(x)
-2.188 -1.188 -0.1875 0.8125 -2.04 2.812 3.812
Pos:761155 = 1
Pos:2155168 = 1
Pos:781687 = 1
Pos:166565 = 0
Pos:830868 = 0

Figure S18

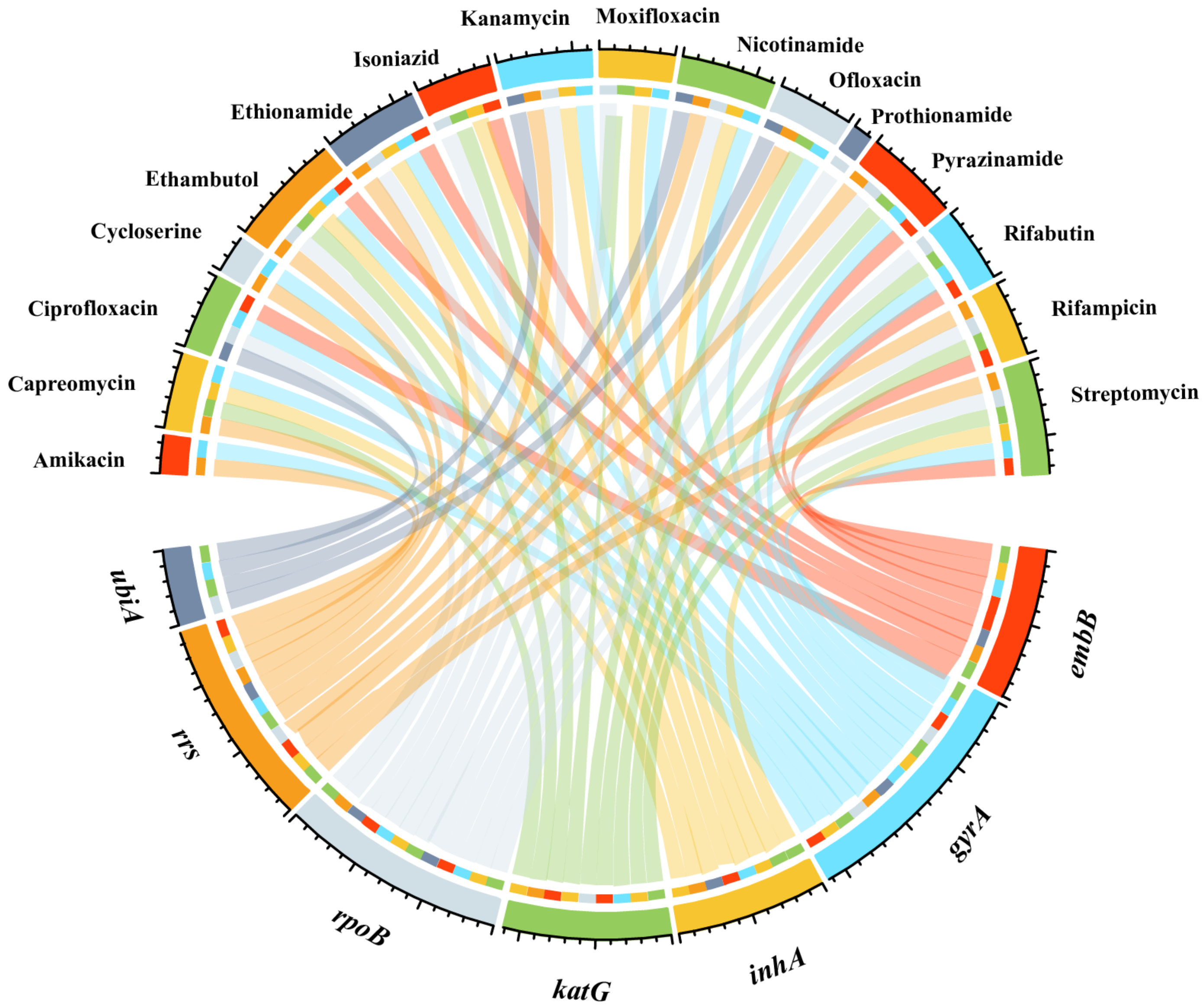
Kanamycin
Moxifloxacin
Nicotinamide
Ofloxacin
Prothionamide
Pyrazinamide
Rifabutin
Rifampicin
Streptomycin
Isoniazid
Ethionamide
Ethambutol
Cycloserine
Ciprofloxacin
Capreomycin
Amikacin
ubiA
rrs
rpoB
katG
inhA
gyrA
embB

Figure S19

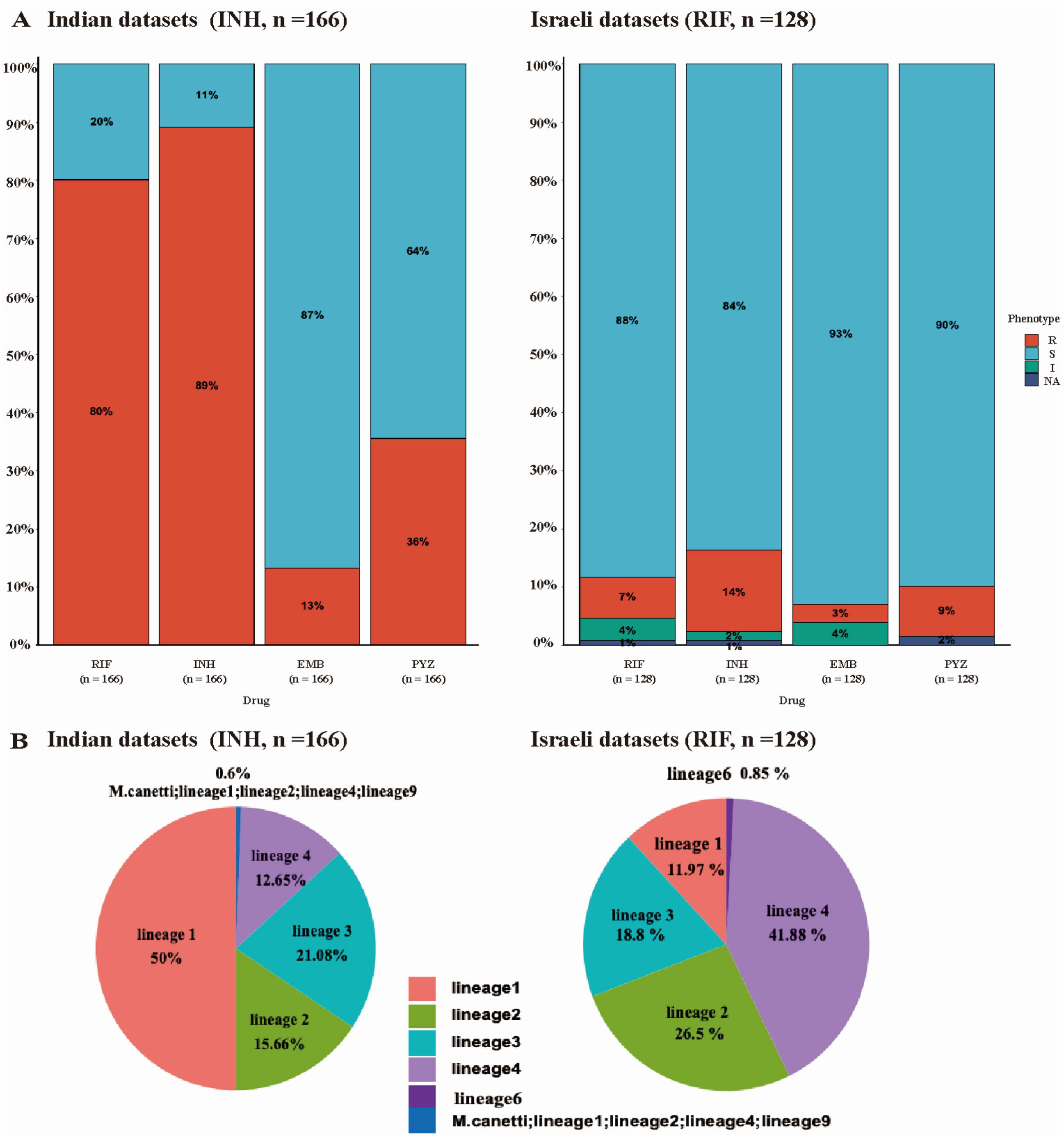

A Indian datasets (INH, n =166)
Israeli datasets (RIF, n =128)
100%
90%
80%
70%
60%
50%
40%
30%
20%
10%
0%
20%
80%
11%
89%
87%
13%
64%
36%
RIF (n = 166)
INH (n = 166)
EMB (n = 166)
PYZ (n = 166)
Drug
88%
7%
4%
1%
84%
14%
2%
1%
93%
3%
4%
90%
9%
2%
RIF (n = 128)
INH (n = 128)
EMB (n = 128)
PYZ (n = 128)
Drug
Phenotype
R
S
I
NA
B Indian datasets (INH, n =166)
Israeli datasets (RIF, n =128)
0.6%
M.canetti;lineage1;lineage2;lineage4;lineage9
lineage 4 12.65%
lineage 1 50%
lineage 3 21.08%
lineage 2 15.66%
lineage6 0.85 %
lineage 1 11.97 %
lineage 3 18.8 %
lineage 4 41.88 %
lineage 2 26.5 %
lineage1
lineage2
lineage3
lineage4
lineage6
M.canetti;lineage1;lineage2;lineage4;lineage9

Figure S20
